# Calcium Involved Directional Organization of Polymer Chains in Polyester Nanogranules in Bacterial Cells

**DOI:** 10.1038/s41598-019-40097-5

**Published:** 2019-03-05

**Authors:** Baoxia Tian, Mohsin Shah, Mun Hwan Choi, Jong Kook Rho, Sang Yeol Lee, Sung Chul Yoon

**Affiliations:** 1 Nano-Biomaterials Science Laboratory, Division of Applied Life Sciences, Gyeongsang National University, Jinju, 52828 Republic of Korea; 2 Faculty of Life Science and Food Engineering, Huaiyin Institute of Technology, Huaian, 23003 People’s Republic of China; 3 Department of Physiology, Institute of Basic Medical Sciences, Khyber Medical University, Peshawar, 40000 Pakistan; 4 Systems & Synthetic Agrobiotech Center, Gyeongsang National University, Jinju, 52828 Republic of Korea

## Abstract

Soil bacteria accumulate polyesters (typically poly([R]-3-hydroxybutyrate (PHB), in which one end of the chain terminates with a carboxyl group) in the form of hydrated, amorphous nanogranules in cells. However, it is not clear what drives the structure of these biomaterials inside bacterial cells. Here, we determined that calcium guides intracellular formation of PHB nanogranules. Our systematic study using the surface zeta potential measurement and the carboxyl-specific SYTO-62 dye binding assay showed that the terminal carboxyl is not exposed to the granule surface but is buried inside native “unit-granules” comprising the mature granule. Extracellular Ca^2+^ was found to mediate the formation of these PHB unit-granules, with uptaken Ca^2+^ stored inside the granules. Comparative [Ca^2+^]-dependent fluorescence spectroscopy revealed that the native granules in *Cupriavidus necator* H16 act as a Ca^2+^ storage system, presumably for the regulation of its cytosolic Ca^2+^ level, but those from recombinant *Escherichia coli* do not. This study reveals intimate links between Ca^2+^ and native granule formation, and establishes a novel mechanism that intracellular PHB granules function as Ca^2+^ storage in order to relieve soil bacteria from Ca^2+^ stress.

## Introduction

Polyhydroxyalkanoic acids (PHAs) are important biomaterials accumulated in the form of discrete cytoplasmic, nanogranular inclusions inside many bacteria and have been suggested to serve as carbon and energy reserves^1,2^. The most abundant PHA is poly([R]-3-hydroxybutyrate) (PHB). About 30 years after its discovery in the 1920s, PHB was recognized as the prototypical biodegradable thermoplastic with the potential to solve the challenge of waste disposal^3^. Since then, there have been remarkable advances in our understanding of PHA metabolism in cells^1–3^, and its application has been extended to the area of specialty materials, such as nanobeads and nanobiomaterials for drug delivery^4–7^.

However, little is known about the structure and assembly of PHA nanogranules in cells^4^. Various techniques, such as electron microscopy, wide-angle X-ray scattering, NMR spectroscopy and confocal microscopy, have been used to investigate the structure of PHA granules. *In vivo* NMR spectroscopy^8^ and X-ray analysis^1^ have shown that PHA in native granules is amorphous, even after isolation. From the crystallization kinetics analysis of medium-chain-length PHA, it was suggested that the polymer is more highly organized in native granules than in organic solvent-reshuffled artificial granules^9^. Analysis of PHA granules by atomic force microscopy (AFM) has shown porin-like structures in the surrounding membrane that are suggested to provide a portal to the amorphous polymer core and to act as sites of PHA metabolism and depolymerization^10^. The studies reported thus far have not provided any specific information about the structure (chain orientation or folding) of the polymer chains in the granules. The polymer chains in native granules have long been assumed to adopt randomly folded (entangled) conformations in an amorphous state of hydrated meta-stable structures^4,8,11^. The characterization of the amorphous structures of water-plasticized, gel-like PHA materials at the molecular or atomic level have long thus been being a challenging problem.

In the present report, we investigated the polymer chain topography in both native and artificial PHB granules using the surface zeta potential, the carboxyl-specific SYTO-62 dye binding assay, divalent cation sorption, and comparative [Ca^2+^]-dependent fluorescence spectroscopy. The surface characterization investigated in this work revealed that the terminal carboxyl groups of PHB chains are not exposed to the surfaces of intracellular PHB granules in *Cupriavidus necator* H16. The [Ca^2+^]-dependent fluorescence spectroscopic data demonstrated that the directional PHB chain orientation may be essential for the calcium storage system in PHB-accumulating soil bacteria that harbor an innate *pha* gene locus^4,12^, which is described in the Results section. For comparison, we also investigated the polymer chain topography of native PHB granules from a recombinant *Escherichia coli* harboring the *phbCAB* operon responsible for PHB synthesis but not phasin gene (*phaP*). The single operon which regulates the three genes i.e *phbCAB*, encode the enzyme PHB synthase (PhbC), β-ketothiolase (PhbA) and NADPH-dependent acetoacetyl-CoA reductase (PhbB), respectively, is involved in the synthesis of PHB inside bacterial cell. PhbC is the key enzyme for PHB biosynthesis, which polymerizes (R)-3-hydroxybutyl-CoA thioester monomers into polymers. PHB can be degraded by depolymerases (PhaZ) intracellularly or extracellularly. PhaP is an amphiphilic protein localized in the interfacial region between cytosolic water and hydrophobic PHB inclusion body and responsible for the regulation of the size and number of PHB granule^4,12^. However, the mechanism of the involvement of phasin protein in the self-assembled formation of PHA granules in the cells is hitherto unknown.

Calcium ions are ubiquitous cations in soil and water and play critical roles in all organisms. Cells invest much of their energy to controlling their Ca^2+^ concentration because Ca^2+^ precipitates phosphate^13^. Hence, cells have evolved ways to sequester this and similar divalent cations, perhaps initially to simply reduce its cytosolic levels but later to use its binding energy for signal transduction. A more plausible explanation for the role of Ca accumulation in the granules is in analogy to the situation of other metal ions such as storage of iron in ferritin during iron-replete conditions and ferritin degradation in low-iron conditions^14^. Therefore, cells must chelate, compartmentalize or extrude this and similar divalent cations. Thus far specific, cellular proteins have been shown to be responsible for these Ca^2+^-controlling activities^15,16^. However, we report here that it is intracellular PHA nanogranules that function as nonproteinaceous Ca^2+^ chelators/containers in soil microorganisms. Our viability study of *C*. *necator* H16 WT and the *Δ phbC* mutant shows that the intracellular PHA nanogranule assembly is an essential element for soil bacteria to survive in the nutrient-poor, Ca^2+^-rich environments.

## Results

### PHB native vs artificial granules

**Figure 1:**
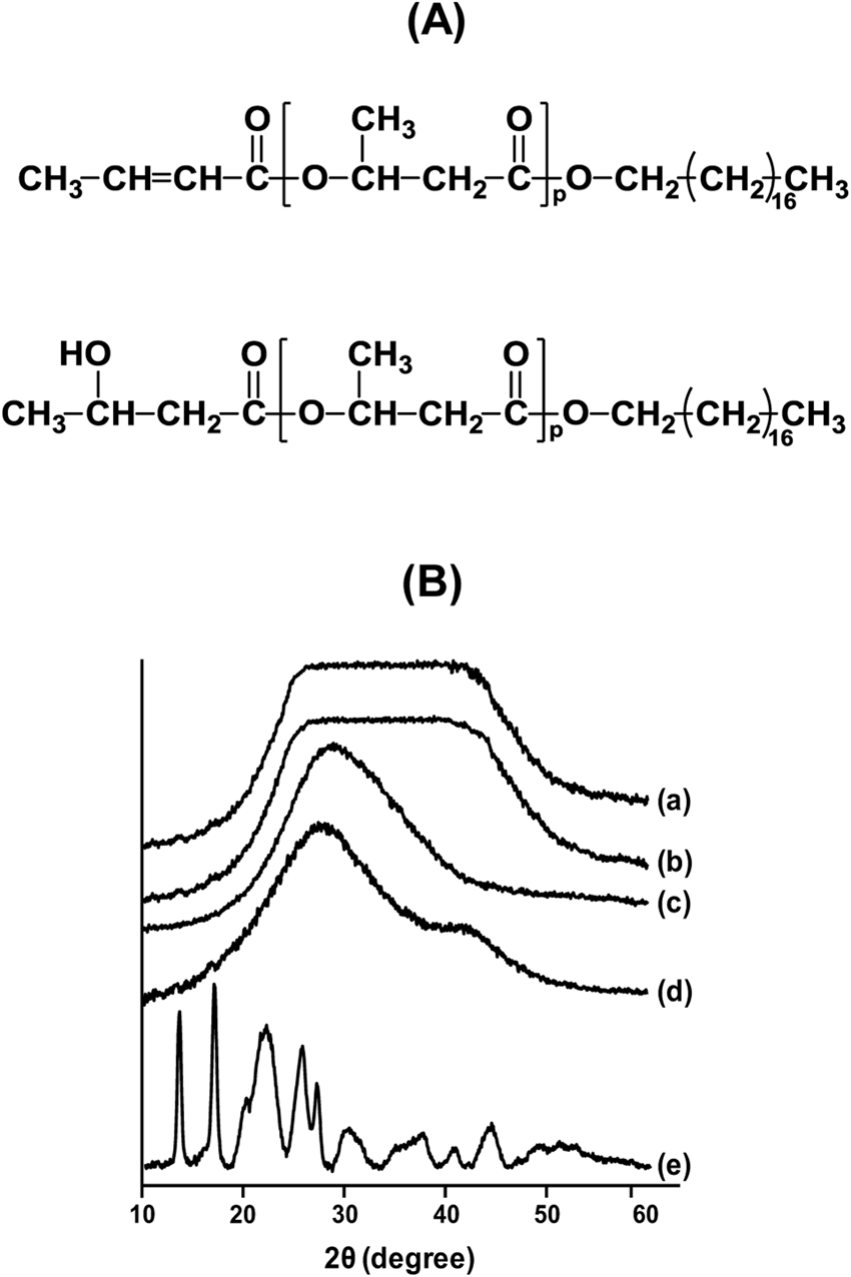
Structural characteristics of granules (**A**) Chemical structure of PHB-1-octadecanol. See Supplementary Fig. S1 for the details. (**B**) X-ray diffractograms. (a) Native PHB granules. (b) Native PHB granules (acetone washed). (c) Artificial PHB granules. (d) PHB-1-octadecanol granules. The polymer granules in (a–d) were suspended in water. (e) Dried PHB-1-octadecanol powder. The X-ray diffractograms are vertically displaced for clear presentation.

We prepared artificial and native PHB granules using chloroform/water emulsion and hypochlorite treatment of the bacterial cells, respectively. When preparing the native granules, only chemical (hypochlorite and acetone)-aided washing was used without any sonication because ultrasonic waves was found strong enough to disintegrate the structure of native granules. The hypochlorite stripping procedure is known to disintegrate the proteins only on the granule surface and to retain the original morphological structures of the PHB granules in the cell^12^. The hypochlorite-processed and acetone-washed PHA granules (hereafter called “native” granules) and organic-solvent-reshuffled “artificial” granules are composed of more than 99 wt% PHB (by elemental analysis) and are free from any proteins or lipids^12^. All of the granules prepared were X-ray amorphous (Fig. 1B).

### Carboxyl end-groups of PHB chains in native granules are not exposed to the surfaces

Here, we hypothesized that the carboxyl end-groups may be buried inside the granules for native granule while in artificial granules, where the polymer chains are randomly reshuffled during the formation process; many terminal carboxyl groups are likely to be exposed to the granule surface during chloroform evaporation. To test this hypothesis, we esterified the terminal carboxyl group of the PHB polymer with 1-octadecanol to synthesize PHB-1-octadecanol (detailed results are provided in Fig. 1A and Supplementary Fig. S1)^17^. PHB-1-octadecanol was used as control molecule throughout the study. Amorphous nature of the PHB-1-octadecanol nanogranules (prepared using a chloroform/water emulsion technique, sized ~200 nm; Supplementary Table S2) suspended in water was confirmed by XRD analysis (Fig. 1B).

**Figure 2:**
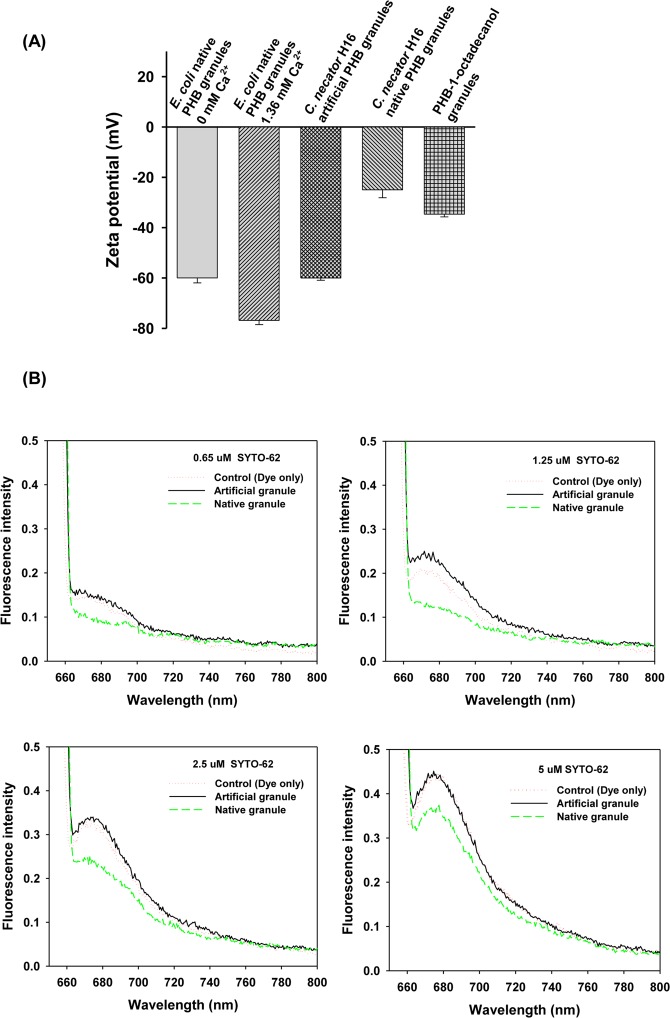
Location of the polymer chain carboxyl end-groups in artificial and native granular PHB. (**A**) Zeta potentials of artificial and native PHB nanogranules and terminal carboxyl-capped PHB-1-octadecanol nanogranules. The PHB granules and polymer were isolated from *C*. *necator* H16 grown with 20 g/L fructose. The artificial PHB and PHB-1-octadecanol nanogranules for the zeta potential measurement were prepared under detergent-free conditions. The data for the native PHB granules obtained from *E*. *coli* harboring the *phbCAB* operon are shown for comparison. Data are represented as the mean ± SD. (**B**) Fluorescent dye adsorption for location of the carboxyl groups in artificial and native granular PHB. The fluorescent dye SYTO-62 was used to stain the surfaces of the PHB granules. The OD value (at 660 nm) of the two granule suspension was 0.6 where the granule size and the amount of PHB were similar.

The hypothesis was confirmed by zeta potential measurements (Fig. 2A). Artificial PHB granules which are not capped with 1-octadecanol, native PHB granules and PHB-1-octadecanol granules (Supplementary Table S2) were found to have zeta potentials of −60.0, −25.0 and −34.7 mV, respectively. The surface of the artificial PHB granules was much more highly negatively charged than that of the native PHB and PHB-1-octadecanol granules. Compared with the zeta potential value of the PHB-1-octadecanol granules, which had low levels of free carboxyl groups (98% esterified, Supplementary Fig. S1), the more positive value for the native granules indicated that almost no free carboxyl groups were present on the surface of the native granules. The location of the carboxyl end-groups was confirmed by fluorescence experiments. The fluorescent dye SYTO-62 can be used to quantify carboxyl groups on the surface of poly (methyl methacrylate) microbeads^18^. The reversible noncovalent SYTO-62 dye adsorption assay is reported to be sensitive enough to determine weak carboxylation. The dye responds to carboxylates but is quenched by hydrophobic interactions. Compared to unbound dye (control), the fluorescence intensity of SYTO-62 bound on the native granules was decreased, whereas the intensity of the dye on the artificial granules was enhanced (Fig. 2B). At 1.25 μM SYTO-62, a much clearer difference between the two granule types was obtained. The equal OD value (at 660 nm) was employed at 0.6 for all the granule samples in Fig. 2B. Further increase in the dye concentration decreased the intensity gap between the control and the granules, resulting in almost overlapping between the control and the artificial granules at 5 μM. The more closely spaced approach to the control values at the high SYTO-62 concentrations must be due to the substantial increase in the concentration of the free, unbound dye molecules, resulting from binding saturation, but the saturation point could be affected by any probable adventitiously bound metal ions from the granule isolation procedures even though we used ultrapure water in the preparations. Thus, the two surface analysis data correlate with each other and support the assumption that the carboxyl end-groups of the PHB chains in *C*. *necator* H16 native granules are not exposed to the granule surface.

### Ca^2+^ is the major cation in native granules

**Figure 3:**
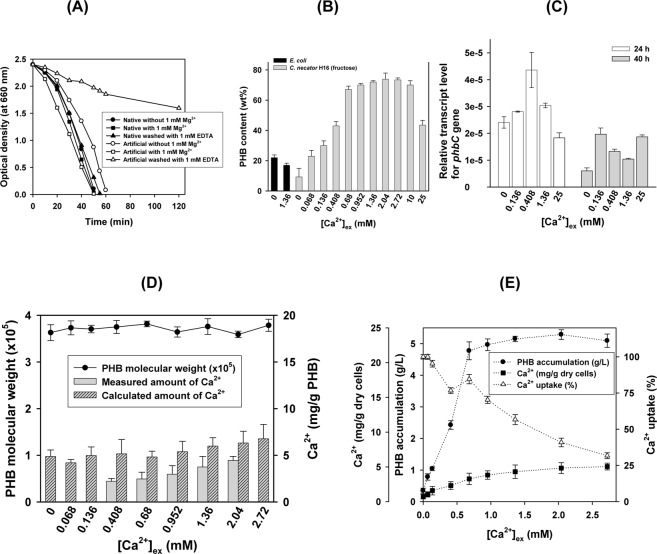
Ca^2+^ as a major cation for terminal carboxyl complexation, its effect on PHB synthesis and its storage in PHB nanogranules. (**A**) Effect of Mg^2+^ (1 mM) and EDTA (1 mM) on the degradation of artificial and native PHB granules by *Pseudomonas stutzeri* BM190 depolymerase. (**B**) Effect of the extracellular Ca^2+^ concentration on PHB accumulation in *C*. *necator* H16 and recombinant *E*. *coli* harboring the *phbCAB* operon. (**C**) Real-time PCR analysis of [Ca^2+^]_ex_-dependent *phbC* expression in H16 WT cells grown on 20 g/L fructose-containing M1 medium. Extracellular Ca^2+^ stimulates *phbC* expression in H16 WT cells. (**D**) Amount of Ca^2+^ in PHB granules in *C*. *necator* H16 grown in M1 medium with 20 g/L fructose in the presence of additional Ca^2+^. The molecular weight of PHB was determined by the viscosity average method. (**E**) Ca^2+^ uptake of PHB nanogranules in the cells grown on 20 g/L fructose. Data are represented as the mean ± SD.

From the above results a divalent cation, such as Ca^2+^ or Mg^2+^, was suspected to be present in the carboxyl end-groups regions located on the inside of H16 native granules. The addition of 1 mM Mg^2+^ caused a significant increase in the enzymatic degradation rate of the artificial granules but not in that of the native granules (Fig. 3A). Washing with 1 mM EDTA significantly decreased the degradation rate of the artificial granules, whereas little effect was observed for the native granules. The inertness of the native granules may indicate the strong association of a divalent cation in the carboxyl end-groups regions. In this regard, we determined the amount of divalent cations in the artificial and native granules using the ICP method. The PHB polymer recovered by CHCl_3_ extraction of *C*. *necator* H16 cells grown in the presence of 1.36 mM CaCl_2_ (the optimum concentration for PHB accumulation) in M1 medium containing 20 g/L fructose had 1.23 mg of Ca^2+^ and 0.03 mg Mg^2+^ per g of PHB. In contrast, in the native counterpart granules, 3.59 mg of Ca^2+^ and 0.05 mg of Mg^2+^ was detected in 1 g of PHB. These findings indicate that Ca^2+^ is the major cation bound to the carboxyl end-groups regions. Other types of structurally different native PHA granules assembled in various soil bacteria were also found to contain calcium ions (Supplementary Table S3).

### Ca^2+^ as a mediator for PHB synthesis and nanogranule formation

To understand the role of Ca^2+^ in PHB granule formation, PHB accumulation in *C*. *necator* H16 cells was investigated in the presence of 0 to 4 mM CaCl_2_ in M1 medium containing 20 g/L fructose as a carbon source. Omission of the additional Ca^2+^ in the PHB synthesis medium containing fructose significantly suppressed the level of PHB accumulation in the cells (dry cell weight, 2.31±0.18 g/L; PHB accumulation, 15 ± 0.3 wt%) (Fig. 3B). For all of the cultures with various amounts of CaCl_2_, the weight of the PHB-excluded dry biomass was 2.00–2.30 g/L. The finding indicates that the additional calcium ions affect PHB synthesis, not cell growth. Furthermore, transfer of the PHB-free cells from medium without calcium to that with calcium led to PHB accumulation in the cells. These observations also indicate that Ca^2+^ is involved in PHB synthesis-associated formation. However, in *E*. *coli* harboring the *phbCAB* operon, PHB accumulation was found to be affected little by additional Ca^2+^ in the medium (Fig. 3B). Furthermore, the native granules from recombinant *E*. *coli* cells grown in the presence of 0 and 1.36 mM Ca^2+^ exhibited zeta potential values of −59.5 and −76.4 mV, respectively, which are close to the value of the artificial H16 PHB granules (Fig. 2A). These observations indicate that PHB granule formation in *E*. *coli* cells is little controlled by Ca^2+^. Therefore, the extracellular calcium ([Ca^2+^]_ex_) dependency was found to be clearly limited to bacteria with an intrinsic PHB accumulation system, including *Pseudomonas* spp. (Supplementary Table S3). However, a much higher increase in [Ca^2+^]_ex_ up to 25 mM resulted in rather low levels of PHB accumulation, which decreased to 43.5 wt%, in H16 WT cells. Thus, as shown in Fig. 3B, [Ca^2+^]_ex_-controlled PHB accumulation played a role only in the low range (0 to 10 mM) of [Ca^2+^]_ex_. To identify the probable functional role of external Ca^2+^ in the enhancement of PHB accumulation, we measured the [Ca^2+^]_ex_-dependent expression of the PHB synthase *phbC* gene, which is responsible for PHB chain elongation, at the growth times of 24 and 40 h, which correspond to the start and mid-exponential phase of PHB accumulation, respectively (Fig. 3C). The real-time PCR data clearly demonstrated that extracellular Ca^2+^ stimulated *phbC* expression in H16 WT cells grown in nutrient-unbalanced M1 medium containing an excess carbon source.

To trace the Ca^2+^ added to the medium, we determined the amounts of Ca^2+^ stored in native PHB granules and that taken up by H16 WT cells grown on fructose (Fig. 3D,E). The amount of Ca^2+^ measured in the native PHB granules was a function of [Ca^2+^]_ex_, ranging between 2.04 and 4.10 mg/g PHB. Assuming the calcium detected in the whole cells is mostly that located in the carboxyl end-groups regions, we calculated the theoretical amounts of Ca^2+^ (mg/g PHB), which ranged between 4.2 and 6.8 mg/g PHB. The experimentally measured values are rather low compared with the calculated values but both depending on [Ca^2+^]_ex_,, suggesting that significant amounts of the uptaken Ca^2+^ ions are encapsulated in PHB granules. The cells with higher levels of PHB accumulation demonstrated a gradual decrease in calcium uptake (%) with increasing [Ca^2+^]_ex_ (Fig. 3E).

### Structure of intracellular amorphous Ca^2+^-associated PHB nanogranules

**Figure 4:**
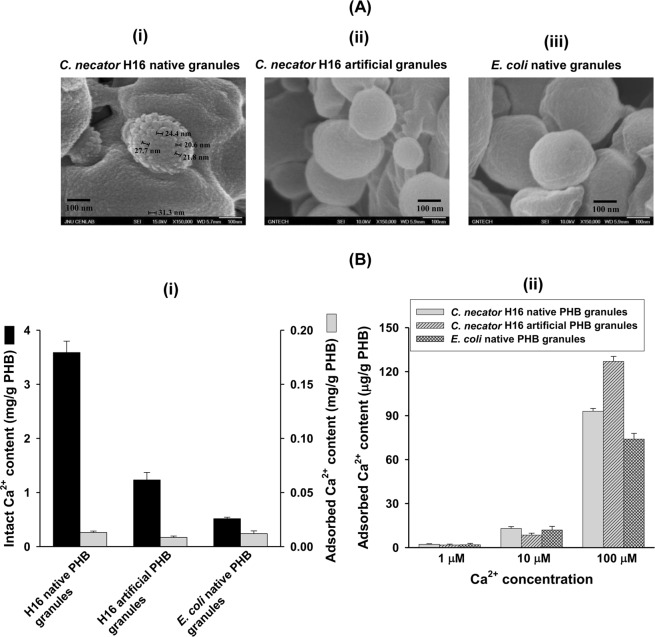
Structural characterization of PHB nanogranules by electron microscopy and metal ion sorption. (**A**) FE-SEM morphology. (i) Acetone-washed PHB nanogranules covered with presumed “unit-granules” sized ~30 nm in diameter. (ii) Artificial PHB nanogranules prepared from the H16 strain. (iii) Acetone-washed *E*. *coli* native PHB granules. The native PHB granules were prepared using a hypochlorite treatment procedure. (**B**) Relative sorption of Ca^2+^ to various PHB granules. The sorption experiment was carried out in distilled water (pH 4.0–4.3) at 30 °C for 5 h. (i) The Ca^2+^ adsorption at [Ca^2+^]_ex_ = 10 μM was compared between the granule samples. (ii) [Ca^2+^]_ex_-dependent Ca^2+^ adsorption of native and artificial PHB granules.

Field emission-scanning electron microscopy (FE-SEM) surface morphology image analysis revealed that acetone washing of hypochlorite-processed *C*. *necator* H16 PHB nanogranules (H16 native granules) exposed small granules sized approximately 20 to 30 nm in diameter with clear phase boundaries among the small granules, referred to as “unit-granules” in this study (Fig. 4A(i)). However, no “unit-granule”-like morphological features were observed for either the artificial H16 (Fig. 4A(ii)) or native *E*. *coli* PHB granules (Fig. 4A(iii)) in this study, suggesting the uniqueness of “unit-granules” only in PHA-accumulating soil bacteria. The presence of separate 20 ~ 30 nm “unit-granules” entities in H16 native granules demonstrates that the PHB chains in the unit-granules are confined to each unit-granule, not stretched and entangled among different granules and thus have finite chain end-to-end distances contrary to the artificial granules with entangled chain morphology. The presence of “unit-granules” in native granules is supported by the recent AFM study of the structures recovered from the sonication of *C*. *necator* H16 cells, which showed ~50-nm globular structures, with each granule containing a ~15-nm central pore^10^.

The polymer chains in native granules have long been assumed to adopt randomly folded conformations in an amorphous state of hydrated meta-stable structures^4,8,11^. However, the randomly folded conformation could not explain all our above results well. Besides, the adsorption of small molecules into large macromolecules usually depends on the geometry of the sorption sites. Therefore, it was speculated that the polymer chains in native granules are assembled in orderly folded conformations. To verify the hypothesis, we performed calcium absorption experiments against all the types of PHB granules. Compared with the Ca^2+^ content of the H16 native granules, the amount of Ca^2+^ adsorbed was very low. Only 1–130 μg of Ca^2+^ per g of PHB was adsorbed depending on the external Ca^2+^ concentration in the range of 1–100 μM. The amount of Ca^2+^ present in the isolated native granules was several hundred-fold higher than that adsorbed *in vitro* (Fig. 4B(i,ii)). The calcium condensation in the carboxyl end-groups regions could be related to the tight regulation (100–300 nM) of intracellular Ca^2+^ in PHB-accumulating prokaryotes^19^. Thus, the very low calcium absorption of the PHB granules at 1 and 10 µM [Ca^2+^]_ex_ implies that, assuming no other calcium-chelating ligand is present, a specific folded conformation of polymer chains in native granules coordinates calcium ions. In fact, the existence of any small calcium-chelating ligand molecules in the granules was found to be unlikely from a detailed HPLC analysis of the enzymatic degradation products (Supplementary Fig. S2). The ester groups on the polymer chain might be the coordination sites for ion-dipole interactions with Ca^2+^ ^20^.

### Divalent metal ion adsorption

As noted earlier, PHB synthesis occurs only in the presence of calcium ions. Thus, without the assumption of a calcium storage system in native granules, no other explanation could account for the higher level of intact calcium in the native granules compared to that adsorbed to the other types of granules tested (Fig. 4B(i,ii)). In addition, in the comparative sorption of *C*. *necator* H16 native and artificial granules against the other four selected divalent metal ions, including Mg^2+^, Ni^2+^, Cd^2+^ and Zn^2+^, the artificial granules displayed a two-fold lower sorption compared with the native granules for Ni^2+^, Cd^2+^ and Zn^2+^ at 10 μM, suggesting a conformational structural difference between these two granule types (Supplementary Fig. S3). All five cations adsorbed to the granules in a quite similar manner depending on their concentrations irrespective of the type of counter anions (chloride for Ca^2+^, Ni^2+^, and Cd^2+^ and sulfate for Mg^2+^ and Zn^2+^). Thus, the sorption mostly occurs nonspecifically and electrostatically. The ester groups of polyester chains could be electrostatic coordination sites of the cations. *C*. *necator* H16 and *E*. *coli* native granules showed quite similar sorption capacity whereas the artificial granules displayed significantly lowered sorption compared to the native granules. The four metal ions Mg^2+^, Ni^2+^, Cd^2+^ and Zn^2+^ exhibited more similar and stronger sorption than Ca^2+^. The 3 to 5 times lower sorption capacity of Ca^2+^ than the other four cations (Supplementary Fig. S3) and the only 1~130 microgram level of Ca^2+^ adsorption per g PHB (Fig. 4B(ii)) may imply that Ca^2+^ would not adsorb to PHB granules nonspecifically but rather well to a storage site with a specific folded conformation in the meta-stable amorphous structure.

### Fluorescence spectroscopic study

**Figure 5:**
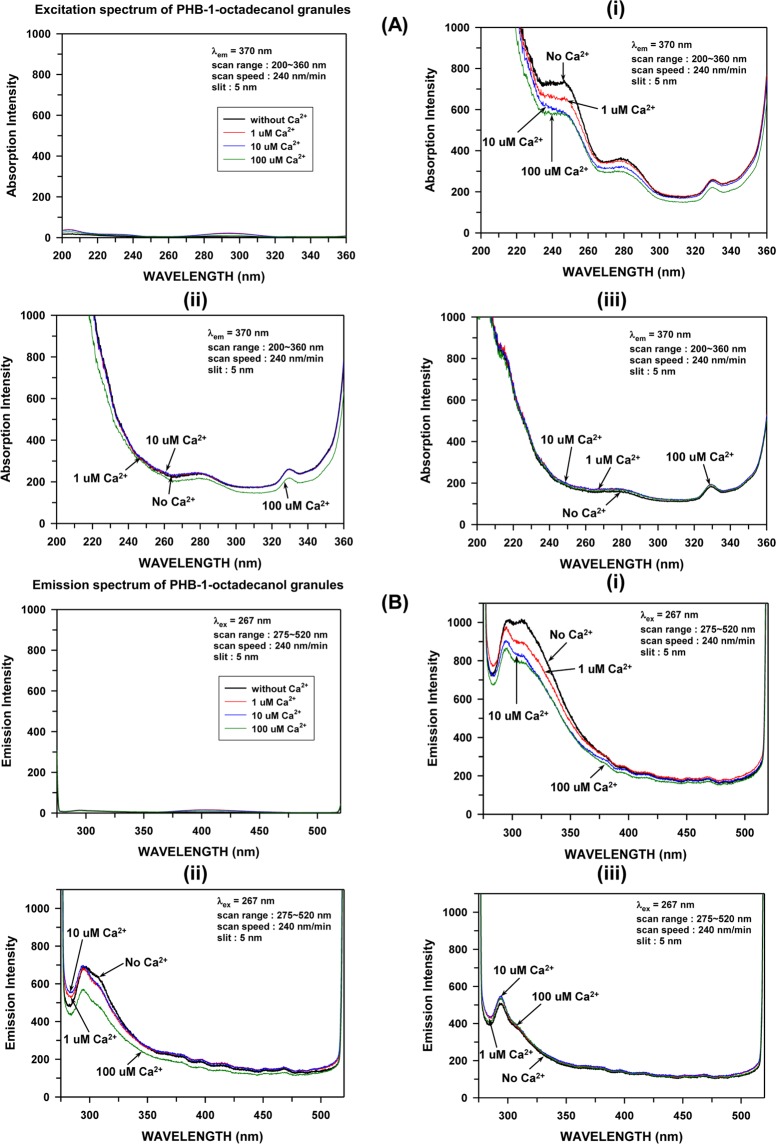
Fluorescence spectroscopy for “Ca^2+^ storage system”: (**A**) Fluorescence excitation spectra of (i) H16 native, (ii) H16 artificial and (iii) *E*. *coli* native PHB granules suspended in water. (**B**) Fluorescence emission spectra of (i) H16 native, (ii) H16 artificial and (iii) *E*. *coli* native PHB granules suspended in water. The excitation and emission fluorescence spectrum of PHB-1-octadecanol granules suspended in water is shown in the left panel in (**A**,**B**), respectively.

The probable structural differences between native and artificial granules^9^ were further investigated using fluorescence spectroscopy. We assumed that calcium ions might contribute to their structural stabilization. Therefore, we added Ca^2+^ at concentrations of 0, 1, 10 and 100 μM to each PHB granule suspension and then incubated them at 30 °C for 5 h. The esterified capping of the carboxyl terminus of the PHB chain with 1-octadecanol led to a complete loss of the absorption and emission signal in both the excitation and emission processes (the left panels in Fig. 5A,B), indicating that the major fluorescence signals of the native and artificial PHB granules originated from the free carboxyl ends (Fig. 5A,B). Comparison of the excitation spectra revealed that the excitation profiles of the native and artificial granules are significantly different, particularly in the region of ~240 nm, whereas the excitation profile of the artificial granules closely resembled that of the recombinant *E*. *coli* native granules (Fig. 5A). The latter finding indicates that the carboxyl terminal domains in the native and artificial PHB granules are located in different molecular environments, clearly demonstrating that the long-range polyester chain arrangements differ between native and artificial granules and that the polymer chains in the *E*. *coli* native granules are organized with random orientations similar to that in artificial granules. The addition of 1 μM Ca^2+^ significantly quenched the excitation fluorescence of the *C*. *necator* H16 native granules in spite of their high intrinsic contents of Ca^2+^ stored, but little effect was observed for the artificial and *E*. *coli* native granules. Even though the exact molecular nature of the fluorescence quenching occurred by Ca^2+^ addition is currently not known, we infer that in native granules external calcium ions may interact sensitively with the buried terminal carboxyl regions inside the unit-granule through a channel, if any. Further addition of Ca^2+^ up to 100 μM also quenched the fluorescence although less sensitively, suggesting a saturated binding phenomenon. Along with the high intact calcium content observed in the native granules (Fig. 4B(i,ii)), the very sensitive fluorescence quenching at only 1 μM Ca^2+^ indicates the presence of a calcium storage system in *C*. *necator* H16 native granules that may be used to regulate the cytosolic free Ca^2+^ level. Free cytosolic Ca^2+^ level in various bacteria are quite low 100–600 nM^19^. As shown in Fig. 4B(ii), the H16 artificial and *E*. *coli* native granules adsorbed Ca^2+^ in a concentration-dependent manner (72 times higher maximal adsorption at 100 μM than at 1 μM for the artificial granules), but little spectral intensity changes were observed. Thus, the Ca^2+^ adsorption in the granules with randomly oriented chains must be mostly nonspecific. The fluorescence emission spectra also showed similar [Ca^2+^]-dependent changes, particularly at ~310 nm, for the native and artificial granules (Fig. 5B(i,ii)). The emission spectra for the artificial granules implied the presence of a residual calcium storage system, which retained Ca^2+^ even after chloroform treatment, a finding that was supported by the substantial amount of Ca^2+^ in the artificial granules (Figs 4B(i) and 5B(ii)). In the *E*. *coli* native granules (Fig. 5B(iii)), the effect of Ca^2+^ addition was not observed in the emission spectra, likewise in the excitation spectra. All of these findings demonstrate that a PHB structure-dependent calcium storage system exists in PHB-accumulating soil bacteria that harbor an innate *pha* gene locus (Supplementary Table S3).

It is worth noting that the little spectral intensity changes of H16 artificial and *E*. *coli* native granules could be ascribable to a rather low availability of free carboxylates because of very likely metals adventitiously bound to the granule surface. However, the sensitive fluorescence change of the H16 native granules may mean that the calcium storage system composed of polymer end carboxylates was well reserved during the preparation process. The polymer chains in native granules are in an amorphous state of hydrated meta-stable structures^4,8,11^. Thus, the native granules contain significant level of hydration water. The external calcium may be easily accessible to the carboxylates in the core of calcium storage system, probably via hydrated channels (i.e., water pockets^8^) or unidentified calcium channel if any.

### Role of Ca^2+^-associated PHB nanogranules as a survival kit

**Figure 6:**
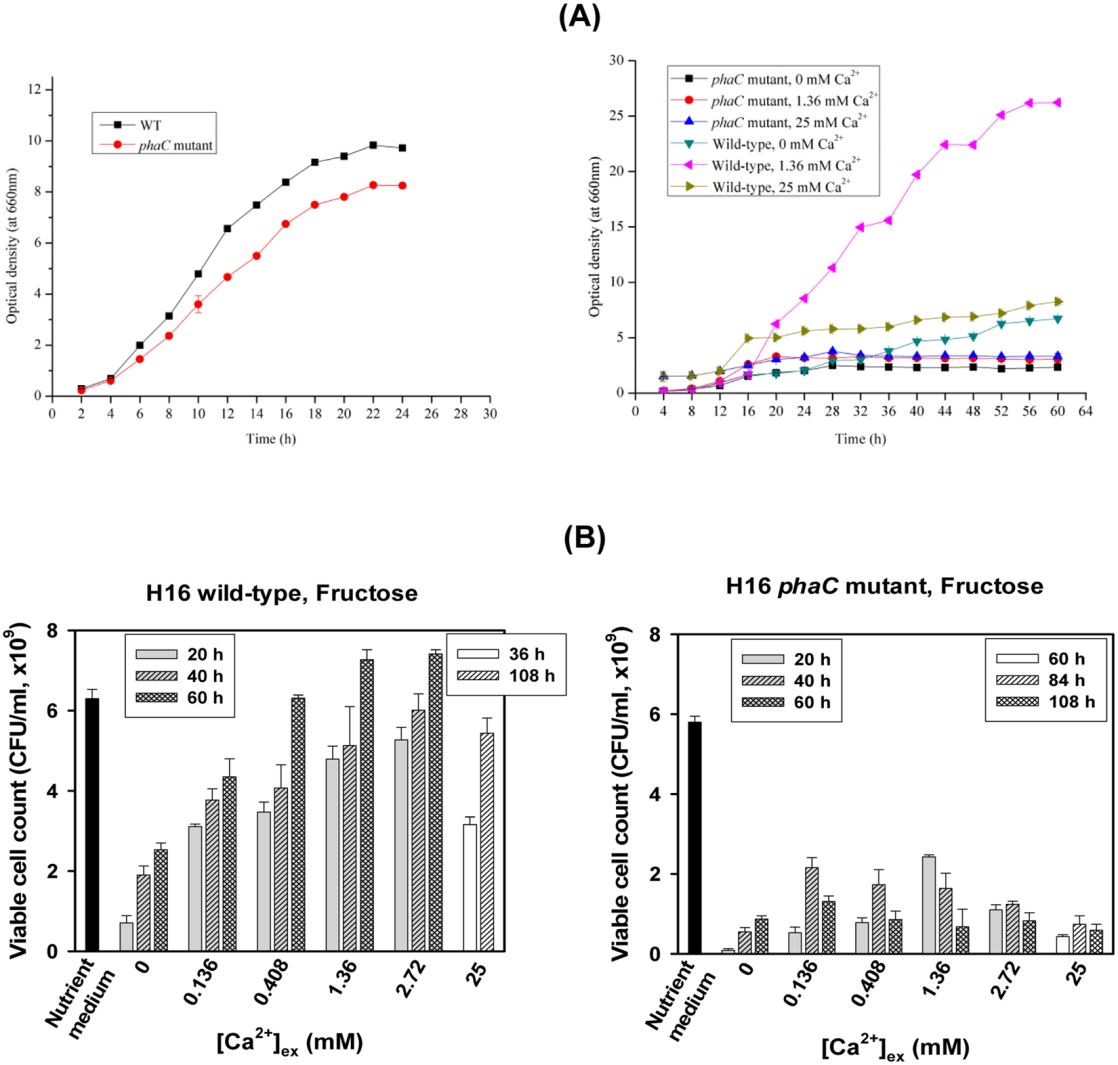
Role of Ca^2+^-associated PHB nanogranules in the survival of *C*. *necator* H16 in nutrient-poor environments. (**A**) Growth characteristics of WT and *phaC* mutant H16 grown in M1 medium with various [Ca^2+^]_ex_ (right) and nutrient broth medium (left). (**B**) Viable cell count of WT and *phaC* mutant H16 grown in M1 medium containing 20 g/L fructose with various [Ca^2+^]_ex_. (Viable cell count data are represented as the mean ± SD).

PHA nanogranular inclusions have been suggested to serve as carbon and energy reserves in the cells of many soil bacteria^1,2^. However, PHA accumulates mostly under unbalanced, nutrient-poor conditions in the presence of an excess carbon source^1,2^. Thus, [Ca^2+^]_ex_-dependent PHB accumulation and Ca^2+^ uptake may have implications for physiological roles that differ from those suggested in the literature. To survive harsh, nutrient-poor environments, soil bacteria may require a special survival kit, particularly in Ca^2+^-rich environments. The different growth characteristics of H16 WT and the *ΔphbC* mutant cells in nutrient-rich medium and nutrient-limited M1 medium (Fig. 6A) clearly support our inference. These cells grew almost equally well in the nutrient-rich medium, but the mutant exhibited poorer growth in fructose-containing M1 medium than the WT over the investigated range of Ca^2+^ concentrations. Furthermore, comparison of the cell viability of the WT and mutant in nutrient broth (NB) and M1 medium in the presence of various amounts of extracellular Ca^2+^ clarified the role of PHB nanogranules, as their viable cell counts were [Ca^2+^]_ex_-dependent when grown with fructose (Fig. 6B). Similar to PHB accumulation, the viable cell count of the WT also increased with increasing [Ca^2+^]_ex_ in the medium (left panel in Fig. 6B), demonstrating a correlation between the PHB accumulation level and cell viability. In contrast, the mutant exhibited approximately 4- to 10-fold lower cell viability without [Ca^2+^]_ex_ dependency (right panel in Fig. 6B). Furthermore, different from the WT, the mutant showed decreased viability later in the growth period (108 h).

### Ca^2+^ associated PHB nanogranules as a cell division effector

**Figure 7:**
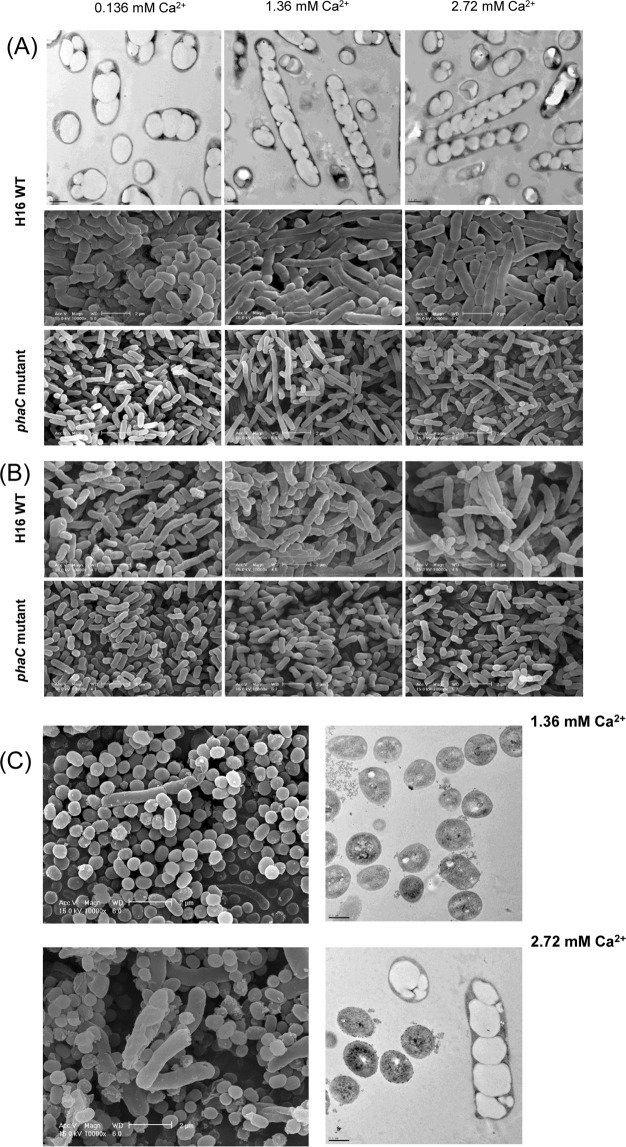
Ca^2+^ associated PHB nanogranules as a cell division effector. FE-SEM and TEM images showing [Ca^2+^]_ex_ dependent elongation of H16 WT and *phaC* mutant cells grown on (**A**), fructose in M1 medium; (**B**), 5 g/L crotonate plus 5 g/L citrate in M1 medium; (**C**), 10 g/L crotonate plus 5 g/L citrate in M1 medium for 108 h. Scale bars: 2 um for SEM images and 0.5 um for TEM images.

Ca^2+^ mediated PHB granule formation was found to affect cell morphology of H16 WT cells depending on [Ca^2+^]_ex_ as shown in the FE-SEM and TEM images in Fig. 7A–C. Cell length of the longest cell (when grown in M1 medium containing fructose) increased up to ~10 µm at the external calcium concentrations higher than 0.68 mM. A rather smaller cell-size increase for the cells grown in minimal medium containing fructose was reported^21^. TEM image analysis for H16 WT cells grown on fructose demonstrated that the cell size increase may be attributed to intracellular Ca^2+^ recruiting caused by the high accumulation of PHB in cells (Fig. 3B and the upper micrographs in Fig. 7A). Thus, at the low range of [Ca^2+^]_ex_ PHB accumulation is [Ca^2+^]_ex_ dependent but at higher [Ca^2+^]_ex_ than 25 mM less PHB accumulation was observed and their cell size and shape were abnormal (data not shown). FE-SEM analysis of the cells grown on crotonate 5 g/L and citrate 5 g/L further confirmed the suggestion (the upper micrographs in Fig. 7B). Such PHB granule formation induced cell elongation was also clearly seen in the cells grown on 10 g/L crotonate and 5 g/L citrate (Fig. 7C). FE-SEM and TEM analysis of the cells grown on crotonate 10 g/L and citrate 5 g/L further confirmed the suggestion. Specifically, at 1.36 mM Ca^2+^, little PHB accumulation was observed in the cells with mostly spherical shapes, but when [Ca^2+^]_ex_ was increased to 2.72 mM, two types of cells spherical and long rod type cells were obtained. Their TEM images revealed that only the long rod type cells were full of mature PHB granules whereas the spherical cells contained few small PHB granules (Fig. 7C). However, both H16 WT and *phbC* mutant cells grown on nutrient-rich media exhibited increased cell size up to ~5 µm independent of [Ca^2+^]_ex_ level in the range of 0~5 mM (data not shown). In addition, the additional Ca^2+^ did not induce significant effect on PHB accumulation (12~15 wt% in cell dry mass) in WT cells as well as the growth of WT and *phbC* mutant cells in NB medium. Thus, the extracellular Ca^2+^ was effective only in nutrient limited M1 medium, not in nutrient rich medium.

## Discussion

An NMR study of PHB *in vivo* suggested that the bulk of PHB in native granules are in a hydrated amorphous meta-stable state most likely because of the plasticizing action of water^8^. Water may either hydrogen bond or form dipole-dipole interactions with the ester groups, thereby inhibiting chain-chain proximity, and localized pockets of water may exist. However, our results shed new light in this regard: the hydrated meta-stable state may be further stabilized by Ca^2+^-coordinated organization, as shown in our [Ca^2+^]-dependent fluorescence quenching experiments. Ca^2+^ is believed to play a role in the stabilization of the meta-stable state of the polymer, but the detailed architecture of the Ca^2+^-containing carboxyl end-group region in a particular conformational state, if any, remains to be identified in terms of a probable Ca^2+^ coordination geometry. Nevertheless, no significant differences in the X-ray diffraction pattern or FTIR spectral bands were observed between the dehydrated samples of native and artificial granules independent of their Ca^2+^ content, suggesting the localization of Ca^2+^ in the carboxyl end-group region so that the Ca^2+^ ions coordination do not significantly affect the overall dimension of the polymer chain folding in the dried state (Supplementary Fig. S4A,B). Furthermore, PHB granules formed in recombinant *E*. *coli* are reported to behave like a semi-liquid^22^ and are most likely structurally similar to *in vitro* synthesized PHB granules^23,24^ and to the H16 artificial PHB granules in this study. [Ca^2+^]_ex_-dependent PHB accumulation and Ca^2+^ storage in the terminal carboxyl-group region of PHB unit-granules in *C*. *necator* H16 clearly indicate that directionally structured PHB granule formation is a Ca^2+^-mediated process. Thus, the complete structural characterization of the Ca^2+^ storage system at the atomic level in a highly mobile state could be a challenging undertaking. However, the storage system may not be molecularly well designed considering the similar Ca^2+^ storage capabilities of various structurally different types of PHA granules (Supplementary Table S3). In addition, all of the genes related to intracellular PHB degradation were found to have no effect on Ca^2+^-controlled PHB accumulation (Supplementary Table S4).

**Figure 8:**
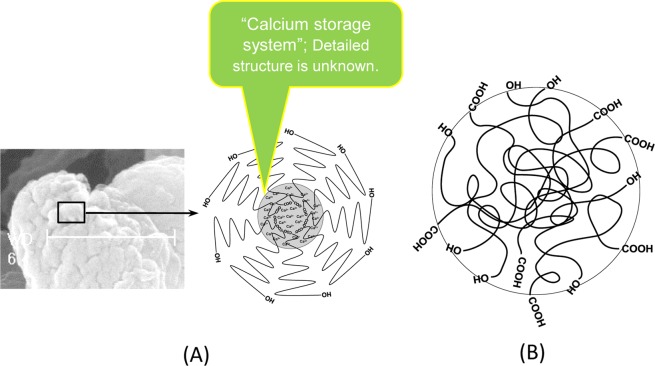
Proposed schematic structural representation of PHB chain rearrangements in native and artificial granules. (**A**) Calcium mediated, directionally controlled PHB chain arrangement in H16 native unit-granule. (**B**) Randomly entangled PHB chain arrangement in H16 artificial granules and *E*. *coli* native granules.

Here, we propose a schematic structural model for the PHB Ca^2+^ storage system in unit-granules (Fig. 8A). Because chain elongation, which is catalyzed by PHB synthase, occurs at the carboxyl end of the growing chain, not at the hydroxyl end^11^, the location of chain growth might be in the unit-granule core. We propose that several synthase enzymes cluster and synchronize the initiation of polymerization, that calcium ions play a role in bundling the carboxyl end-groups and that the polyester chains elongate directionally and radially to form ~30-nm unit-granules with their hydroxyl termini directed outward. In this regard, phasin proteins^4,10,20^ may have certain roles in the directional arrangements of PHB chains as evidenced by all the data in this study for the native granules from recombinant *E*. *coli* harboring the *phbCAB* operon but devoid of *phaP* gene. The central pore observed by Dennis *et al*.^10^ might be related to the Ca^2+^ storage system in the unit-granule core that we have suggested. Thus, Ca^2+^ is considered to stabilize the micelle-like structure of unit-granules. Eventually, the unit-granules assemble to form larger mature granules, sized ~200–500 nm, in the cell.

Another example of Ca^2+^ complexed PHB is the short-chain PHB-polyphosphate-Ca^2+^ (cPHB) widely found in both prokaryotes and eukaryotes, where Ca^2+^ is known to be complexed with polyphosphate^25^. In *E*. *coli*, it acts as voltage-gated Ca^2+^ channels with complex gating kinetics, their activity being implicated in the phase of growth-dependent control of cytosolic Ca^2+^ levels^26^. *E*. *coli* do not harbor the PHB synthesis genes such as *phaC*, *phaP*, *phaZ*, *phaR*, etc. but recently, a periplasmic protein, YdcS has been reported to exhibit short-chain PHB synthase activity^27^. On the contrary, in the case of granular PHB in *C*. *necator* H16, Ca^2+^ is considered to be complexed to the terminal carboxyl groups if we assume there is no other chelating agent such as cyclized 3HB dimer (Supplementary Fig. S2). At least polyphosphate is not involved in the Ca^2+^ complexation in PHB unit-granules because phosphorus was detected little in the native granules which are composed of more than 99% PHB (Supplementary Table S5) and polyphosphate is present as separate granule entities in H16 cell^28^. In PHB accumulating bacteria, the Ca^2+^ containing carboxyl end-group region can be considered as a factory for PHB synthesis and unit-granule assembly. However, the detailed molecular architecture of the Ca^2+^ containing core region remains to be identified. Ca^2+^ is implicated in the regulation of a wide variety of cellular processes, including signal transduction in bacteria, where cytosolic Ca^2+^ levels are tightly controlled by mechanisms involving plasma membrane transporters^16,29^. This study shows that extracellular Ca^2+^ controls PHB synthesis through an unidentified system. Similarly, cPHB production depends on the level of extracellular Ca^2+^ ^29^. Thus, the initial PHB synthesis event may occur via a Ca^2+^ mediated signaling process which is totally unknown.

As shown in the comparison of cell viability between H16 WT and *phaC* mutant grown in nutrient-limited M1 media, PHB nanogranules function as a survival kit in nutrient-poor environment as well as Ca^2+^ container to survive the external calcium stress. Most ecological niches in nature are believed to be nutritionally deficient. Ubiquitous environmental Ca^2+^ could be a stress to soil bacteria so that they may need the intracellular Ca^2+^ masking system for survival and better adaptability to any nutrient-poor natural environment but in nutrient-rich environments require it less critically (a plasticity phenomenon).

During bacterial cell division, FtsZ undergoes guanosine 5′-triphosphate (GTP)-dependent polymerization to form the Z ring at the mid-cell^30,31^. UgtP which localizes to the division site in a nutrient-dependent manner directly inhibits FtsZ assembly^31^. Moreover, uridine-5′-diphosphoglucose (UDP-glucose) is the substrate of UgtP. It is likely that changes in UgtP activity could be caused by the higher levels of UDP-glucose resulting from the use of sugars such as fructose as carbon source in the cultivation of H16 strains. Thus, cell elongation of H16 WT and *phaC* mutant grown on nutrient rich or fructose fed M1 medium may result from the inhibition of FtsZ polymerization by UgtP. But the cell elongation of WT grown on crotonate containing nutrient limited medium is believed to be caused by Ca^2+^ level regulation by PHB nanogranules because *phaC* mutant did not elongate. *In vitro* studies showed that Ca^2+^ stimulates FtsZ polymerization *in vitro*^19,30,32^. PHB granules from crotonate-grown cells contained much more significant amounts of Ca^2+^ (up to ~50 mg/g PHB), probably causing more significantly lowering cytosol Ca^2+^ level and eventually inhibiting cell division. Thus, intracellular PHB nanogranules may control the cytosol Ca^2+^, affecting cell division.

## Conclusions

Calcium plays a pivotal role in numerous biological processes. The regulation of cytosolic concentrations of calcium is essential for the survival of an organism. We infer that the directional PHB chain organization occurs in a limited cytosol space in Ca^2+^ controlled manner in order that the water insoluble hydrophobic polymer chains do not perturb the other cellular system during the formation of unit-granules. The revealed structural difference between native and artificial granules explains the reason why they exhibit very different susceptibility to enzymatic hydrolysis. The Ca^2+^ storage is believed to relieve the cell from Ca^2+^ stress, especially in soil bacteria for survival in nutrient-poor ecological niches in nature. Thus, on the basis of the findings in this study, the role of intracellular PHB granules should be reassessed in terms of cell viability of soil bacteria in Ca^2+^ rich natural environment from the ecological point of view. The authors assure that the Ca^2+^ related results could be exploited in the application studies such as high PHB production in bacterial and plant cells as well as in an enzyme reactor and the design and preparation of novel PHB based nanoparticles and drug implants for biomedical applications with Ca^2+^ controlled polymer chain orientation. The isolated native PHA granules can be used as a storage/transport vehicle for calcium (PCT patented). In addition, ester capping of the carboxyl end group in PHA could be a means to control the degradation rate of PHA bioplastics in nature (US patent application).

## Materials and Methods

### Preparation of native PHA granules

PHA-accumulated cells were harvested by centrifugation (4,000 × g, 7 min), 15 g of wet cells were suspended in 100 mL of cold PBS buffer (8 g/L NaCl, 0.2 g/L KCl, 3.6 g/L Na_2_HPO_4_·12H_2_O, 0.24 g/L KH_2_PO_4_, pH 7.4) and lysed with 6% (w/v) of sodium hypochlorite solution (Duksan Pure Chemicals Co., Ltd., Ansan, Korea) for 8 h under rapid stirring at 4 °C cold chamber. White granules were harvested by centrifugation (6,000 × *g*, 5 min) and washed twice with cold PBS buffer. Granules were suspended in deionized distilled water and then dialyzed with 12~14 kDa CelluSep (Membrane Filtration Products, Inc., Texas, USA) T4 molecular weight cut off (MWCO) cellulose tubing membrane for 2 days against at least 3 times change of deionized distilled water. The recovered PHA granules were stored at 4 °C. The 6% (w/v) hypochlorite solution contained 0.015 mg/L Ca^2+^. The ICP detected Ca^2+^ in the stripping medium(5 mL) was calculated to occupy only 1/1000~1/10000 of the amounts of Ca^2+^ detected in PHB granules recovered, negligible to our analysis purpose.

### Preparation of artificial PHA granules

Artificial PHA granules were prepared by a previously described method^33^ with slight modification. PHB homopolymer (50 mg), solubilized in 1 mL of chloroform was added to 5 mL of aqueous solution of 0.05% (w/v) sodium deoxycholate. The mixtures were emulsified by sonication and the chloroform solvent of emulsified mixtures was removed by centrifugation (2,500 rpm, 3 min) and subsequent stirring in the fume hood for 1 h. After removal of the solvent, the stable artificial PHA granules were obtained.

### Enzyme isolation and purification

*Pseudomonas stutzeri* BM190, isolated in author’s lab, was used as source strains for PHB depolymerases as described in the Supplementary materials and methods.

### Enzymatic degradation of PHA granules

The activity of PHB depolymerase was assayed spectrophotometrically by measuring the initial decrease in the optical density (O.D.) of the PHA granules at 660 nm as detailed in the Supplementary materials and methods.

### Construction of insertional mutants

Amplified internal fragments from the *C*. *necator* H16 *phaC* gene was cloned into the kanamycin-resistant plasmid pVIK112 and introduced into *E*. *coli* S17-1 λ-pir by electroporation^34^. Conjugation was performed by filter mating with *E*. *coli* S17-1 λ-pir (pVIK112-*phaC*) and *C*. *necator* H16 as donor and recipient, respectively. The transconjugant (*C*. *necator* H16 pVIK112-*phaC*) was selected on LB medium containing kanamycin at 30 °C. The other mutants were prepared similarly. All primers’ sequences were provided in the Supplementary Table S1.

### Field emission scanning electron microscopy (FE-SEM)

*C*. *necator* H16 cells were fixed and dehydrated as described previously^35^. Hypochlorite treated PHB granules, isolated from *C*. *necator* H16 cells, were soaked in pure acetone for 1 h and the isolated granules were washed with fresh acetone. All the samples were coated with gold and observed through Philips XL30 S FEG field emission scanning electron microscopy (FE-SEM, Amsterdam, Netherlands).

### Transmission Electron Microscopy (TEM)

*C*. *necator* H16 wild-type cultures grown on PHA synthesis medium were harvested by centrifugation (4,000 × *g*, 7 min) and cells were prefixed in a 2.5% glutaraldehyde solution for 2 h. The samples were washed twice with 0.2 M PBS buffer (pH 7.2) and then postfixed in 1% osmium tetraoxide (OsO_4_) for 1 h. They were washed twice with PBS and dehydrated by exchange with a graded series of ethanol solutions, followed by propylene oxide. Pellets were infiltrated in a 3:7, 5:5, and 7:3 ratios of epoxy resin mixture and propylene oxide each for 1 h and finally embedded in fresh epoxy resin mixture overnight and then polymerized for 48 h at 50 °C. The polymerized blocks were sectioned with ultramicrotome (LEICA EM UC6). The thin sections were stained with uranyl acetate and lead citrate solutions, followed by imaging in a model Tecnai 12 transmission electron microscope (FEI Co., Hillsboro. Oregon, USA) operated at 120 kV in the bright field mode.

### Wide-angle X-ray diffraction (WAXD) analysis

The X-ray analysis of artificial and native, granule suspensions was performed as wet state by using glass capillary. WAXD patterns of the PHA granules were recorded in a General Area Detector X-ray Diffraction System (Bruker AXS, Massachusetts, USA) equipped with a collimator having the diameter of 0.2–0.5 mm. Cu Kα (*λ* = 1.54056 Å) radiation was utilized for all X-ray experiments scanned from 5 to 80° (2 Å) with a step length of 0.05°.

### Zeta potential measurement

The surface charge of the PHA granules was characterized in terms of the zeta potential, which was determined using electrophoretic light scattering (ELS-Z; Otsuka Electronics Co., Osaka, Japan) at a scattering angle of 20°. Native PHB granules were washed to remove any lipid impurities via immersion in acetone for 30 min at room temperature, followed by washing three times with water. Artificial PHB granules and PHB-1-octadecanol granules for zeta potential measurement were prepared under detergent-free conditions. The mean zeta potential of the PHA granules was determined in duplicate, and the average values were calculated.

### Fluorescence SYTO-62 dye adsorption assay

The carboxyl groups on the surface of PHA granules were characterized by fluorescence dye adsorption assay using SYTO-62 (Invitrogen Co., California, USA) and F-7000 fluorescence Spectrometer (Hitachi Co., Tokyo, Japan). The native PHB granule was washed with acetone to remove lipid impurities by immersion for 30 min at room temperature, followed by water washing three times. Artificial PHB granule was prepared under detergent free condition. PHA granules and SYTO-62 dye were reacted in deionized water at room temperature for 1 h and then analyzed. The OD value (at 660 nm) of the two granule suspension was 0.6 where the granule size and the amount of PHB were similar. The dye concentrations were 0.65, 1.25, 2.5, and 5.0 μM. The fluorescence run conditions were as follows: excitation wavelength, 649 nm; emission start wavelength, 650 nm; emission end wavelength, 800 nm; scan speed, 240 nm/min; excitation slit, 5 nm; emission slit, 5 nm.

### Determination of the amount of Ca^2+^ in *C. necator* H16 cells and PHB granules

The amount of divalent cations present in the freeze-dried *C*. *necator* H16 cells, native PHB granules, and chloroform-extracted PHB homopolymers was determined after acid hydrolysis. Each 100 mg of the freeze-dried *C*. *necator* H16 cells or 80 mg of the native PHB granules and chloroform-extracted PHB homopolymers was hydrolyzed with 10 mL of 6 N HCl (Merck, Frankfurt, Germany) for 6 h at 100 °C. Blank aqueous 6 N HCl solution (HPLC grade) was used as a control. Ultra-pure water, 18 MΩ·cm deionized, was used in all experimental steps including cell washing. The concentration of each ion in each aliquot was then determined by inductively coupled plasma-atomic emission spectrometer (ICP-AES) (OPTIMA 5300 DV, Perkin Elmer) analysis. All experiments were performed in triplicate.

### Quantitative real-time PCR analysis of *phaC* gene expression

*C*. *necator* H16 wild-type cells grown on PHA synthesis medium containing 20 g/L of fructose as the sole carbon source and 0 ~25 mM CaCl_2_ were harvested after 24 and 40 h. RNA isolation was performed by using the RNeasy mini kit (Qiagen Co., Hilden, Germany) according to the manufacturer’s protocol. RNA molecules were incubated with DNase I to remove genomic DNA. One-step RT PCR was performed by using Rotor-Gene SYBR Green RT-PCR kit (Qiagen Co., Hilden, Germany) according to the manufacture’s protocol. 16 S rRNA was used as an internal standard to estimate the relative expression level of the target gene by 2^−ΔΔCT^ method^36^. All experiments were performed in triplicate.

### Divalent metal ion adsorption

To evaluate the metal ion binding capacity of PHB granules each 10 mL of native granule (185.6 mg/L) and artificial granule (188.2 mg/L), *E*. *coli* native granule (184.6 mg/Ll^−1^) solutions containing 100 μM, 10 μM, and 1 μM of either Ca^2+^ (as CaCl_2_), Mg^2+^ (as MgCl_2_), Zn^2+^ (as ZnSO_4_), Ni^2+^ (as NiCl_2_) or Cd^2+^ (as CdCl_2_) was incubated at 30 °C, 100 rpm for 5 h. Native PHB granules were washed with acetone to remove lipid and lipophilic impurities by immersion for 30 min at room temperature, followed by water washing three times. Artificial PHB granule was prepared under detergent free condition. After reaction, PHB granules were removed by centrifugation and the concentration of the remaining ion in each clear supernatant solution was then determined by inductively coupled plasma-atomic emission spectrometer (ICP-AES) (OPTIMA 5300 DV, Perkin Elmer) analysis. All experiments were performed in duplicate.

$$ \% \,{\rm{Adsorption}}=({{\rm{C}}}_{{\rm{o}}}-{{\rm{C}}}_{{\rm{e}}}){/{\rm{C}}}_{{\rm{o}}}\times {\rm{100}}$$

where C_o_ and C_e_ are the initial and remaining concentration of metal ions in solution.

### Fluorescence spectroscopy for “Ca^2+^ storage system” study

Each 10 mL of native granule (185.6 mg/L), artificial granule (188.2 mg/L), *E*. *coli* native granule (184.6 mg/L), and PHB-1-octadecanol granule (186.4 mg/L) solutions containing 100 μM, 10 μM, or 1 μM of Ca^2+^ (as CaCl_2_) was shaken at 30 °C, 100 rpm for 5 h. Fluorescence impurities in the deionized water were removed by the treatment of charcoal. Emission and excitation of Ca^2+^ treated PHB granule suspensions were determined by F-7000 FL fluorescence spectrometer (Hitachi, Tokyo, Japan) analysis. The excitation spectra were obtained in the scan range between 200 and 360 nm with the emission at 370 nm, and the emission spectra between 275 and 520 nm with the excitation at 267 nm. The excitation and emission slit widths were set to 5 nm.

### Determination of cell viability

Aliquots of *C*. *necator* H16 wild-type and *phaC* mutant cultures grown on nutrient-rich (NR) or PHB synthesis media were taken at the sampling times of 20, 40, 60, 84 and 108 h, gradient diluted with 0.85% NaCl and subsequently plated onto NR agar plates. All agar plates were incubated at 30 °C, and colony-forming units (CFU) were counted at 36 h. All experiments were performed in triplicate. The PHB synthesis medium contained 37 mM phosphate. The final pH of the cultures grown on fructose was 6.2–6.5 for 0–10 mM Ca^2+^ and 6.0 for 25 mM Ca^2+^, where the initial pH was 7.0.

### Nucleotide sequence accession number

The GenBank accession number for the PHB depolymerases of *Pseudomonas stutzeri* BM190 sequence is EU887946 and *C*. *necator* H16 *phaC* gene is NC008313.

## Supplementary information

Supplementary Information

## Data Availability

Available from the corresponding author on reasonable request.
