# Supplementary material for: Calcium Involved Directional Organization of Polymer Chains in Polyester Nanogranules in Bacterial Cells
Source: Sci Rep. 2019 Mar 5;9:3429. doi: 10.1038/s41598-019-40097-5 (PMC6401383; doi:10.1038/s41598-019-40097-5)
Supplement: Supplementary file 1 — Supplementary Information [file 41598_2019_40097_MOESM1_ESM.pdf]

Supplementary Materials

Calcium Involved Directional Organization of Polymer Chains in Polyester Nanogranules in Bacterial Cells

Baoxia Tian[#,1,2], Mohsin Shah[#1,3], Mun Hwan Choi[#,1], Jong Kook Rho[1], Sang Yeol Lee[4], Sung Chul Yoon[1,4,*]

Supplementary Materials and Methods……………………………………….….pp. 2-10

Supplementary Figures …………………………………………………………pp. 11-19

Figure S1……………………………………………………………………….….p.11-12

Figure S2…………………………………………………………………………..p.13-14

Figure S3……………………………………………………………………….…pp.15-16

Figure S4………………………………………………………………………pp.17-18

Supplementary Tables ……………………………………………………………pp. 19-22

Table S1 …………………………………………………………………………p. 19

Table S2 …………………………………………………………………………p. 20

Table S3 …………………………………………………………………………p. 21

Table S4 …………………………………………………………………………p. 22

Table S5 …………………………………………………………………………p. 23

SUPPLEMENTARY MATERIALS AND METHODS

Reagents. Restriction enzymes and T4 ligase were purchased from New England Biolabs (USA). Go Taq® DNA polymerase, dNTP mixture, and SDS-PAGE molecular standard marker were purchased from Promega (USA). Ni-NTA Chelating Agarose CL-6B was purchased from Peptron (Korea). Yeast extract, nutrient broth, and tryptone were purchased from Difco (Detroit, USA). γ-Butyrolactone, γ-valerolactone, and bis(2-ethylhexanoate) tin were purchased from Sigma-Aldrich Korea Ltd. Glucose, $MgSO_4 \cdot 7H_2O$, $NaHCO_3$, $CaCl_2 \cdot 2H_2O$, $Fe(NH_4)_2 \cdot$citrate, KH_2PO_4, $Na_2HPO_4 \cdot 12H_2O$, $ZnSO_4$, methanol, and chloroform (HPLC grade) were purchased from Junsei Chemical Co. (Japan). 1-Octadecanol was purchased from Tokyo Chemical Industry Co. (Japan). HCl and H_2SO_4 were purchased from Merck (Germany).

Bacterial strains and culture media for PHA accumulation. *Cupriavidus necator* (formerly *Wautersia eutropha*) H16 ATCC 17699 and *Hydrogenophaga pseudoflava* ATCC 33668 were purchased from the American Type Culture Collection and *Paracoccus denitrificans* KCTC 2530 was purchased from the Korean Culture Type Collection. *E. coli* DH10B : pBAD-Ae-pha [1] and *E. coli* DH10B : pMMB-pha [2] were obtained from Prof. S.W. Kim (Gyeongsang National University, Korea). Nutrient-rich (NR) medium was used in the seeding, maintenance, and storage of the three strains and contained 1% yeast extract, 1.5% nutrient broth, and 1% ammonium sulfate. *E. coli* was grown in Luria-Bertani (LB) medium containing 0.5% yeast extract, 1% tryptone, and 1% NaCl. The medium used for PHA accumulation was a modified PHA synthesis medium [4] containing carbon sources. The composition of the PHA synthesis medium was as follows: 2.3 g/L KH_2PO_4, 7.3 g/L$Na_2HPO_4 \cdot 12H_2O$, 1.0 g/L $(NH_4)_2SO_4$, 0.4 g/L$MgSO_4 \cdot 7H_2O$, 0.4 g/L $NaHCO_3$, 0.2 g/L $CaCl_2 \cdot 2H_2O$, 0.03 g/L $Fe(NH_4)_2 \cdot$citrate, and 2 mL/L microelement solution. The pH of the medium was adjusted to 7.0. The microelement

solution contained 0.29 g of $ZnSO_4 \cdot 7H_2O$, 1.98 g of $MnCl_2 \cdot 4H_2O$, 2.81 g of $CoSO_4 \cdot 7H_2O$, 0.17 g of $CuCl_2 \cdot 2H_2O$, 0.3 g of H_3BO_3, 0.03 g of $Na_2MoO_4 \cdot 2H_2O$, 0.02 g of $NiCl_2 \cdot 6H_2O$, 2.78 g of $FeSo_4 \cdot 7H_2O$ per liter of 0.5 N HCl. All growth experiments for PHA accumulation were performed under aerobic conditions in a temperature-controlled shaker (Korea Instrument Co., Seoul, Korea).

Culture conditions. *C. necator* H16 was cultivated for PHB homopolymer accumulation in PHA synthesis medium containing 20 g/L of fructose as the sole carbon source for 72 h at 30℃. *H. pseudoflava* was cultivated to accumulate P(HB-*co*-HV) copolymers in PHA synthesis medium containing an appropriate amount of γ-valerolactone plus 10 g /L of glucose as carbon sources for 72 h at 30℃. PHV homopolymer was accumulated in *P. denitrificans* grown on PHA synthesis medium containing 10 ml/L of *n*-valeric acid as the sole carbon source for 60 h at 30℃. For the PHB accumulation in *E. coli*, two-step cultivation was carried out. *E. coli* DH10B:pBAD-Ae-pha (*C. necator* PHA synthesis operon) and *E. coli* DH10B:pMMB-pha (*Acinetobacter* PHA synthesis operon) were cultivated in 500 mL of LB medium, respectively, and after 12 h, the cells were transferred to 500 mL of 10 g/L glucose containing LB medium and cultivated for 48 h at 37°C. One hundred μg/mL of ampicillin and 34 μg/mL of chloramphenicol were added to the medium for the maintenance of plasmids pBAD-Ae-pha and pMMB-pha, respectively. To investigate the roles of Ca^{2+} in *C. necator* H16, *C. necator* H16 cells were cultivated in NR medium or PHA synthesis medium containing 20 g/L of fructose as the sole carbon source or 5 g/L of citric acid and 5 g/L of crotonic acid as the cosubstrate in the presence of 0 ~ 25 mM $CaCl_2$ at 30℃.

PHA isolation and characterization. For the extraction of PHA, the cells were harvested by centrifugation (4,000 x *g* for 7 min), washed with methanol, and dried overnight under a

vacuum at ambient temperature. PHA was extracted from dried cells with hot chloroform in a Pyrex Soxhlet apparatus for 6 h. The solvent extract concentrated by vacuum rotary evaporation was precipitated in rapidly stirred 10 volumes of cold methanol. The isolated PHA was purified by reprecipitation and was dried overnight under a vacuum at ambient temperature. Quantitative determination of the monomer units in PHA was determined by analyzing the methyl esters, which were recovered from a sulfuric acid/methanol treatment of the PHA, using a Hewlett-Packard HP5890 Series II gas chromatograph (GC) equipped with a HP-1 capillary column and a flame ionization detector (7, 8). A typical GC run condition was as follows: initial temperature 80℃, 2 min; heating rate, 8℃/min; final temperature 180℃, 0.5 min; carrier (He) flow rate, 3 mL/min; injector temperature, 230℃; detector temperature, 280℃. The standardization of each GC peak was made against the PHA of known structure characterized by quantitative nuclear magnetic resonance (NMR) analyses [3, 4]. The ^{1}H-NMR analyses of PHA samples were carried out on a Bruker-DRX 500 MHz spectrometer (Germany). The spectra of the samples were recorded at room temperature in $CDCl_3$. The integration of the split spectral signals was performed with standard software.

Enzyme isolation and purification: *P. stutzeri* BM190 was precultured in NB medium for 12 h, enzymes were obtained by growing the precultured cells in PHB degradation medium (pH 7.5) consisted of 1.7 g/L KH_2PO_4, 6.2 g/L$Na_2HPO_4 \cdot 12H_20$, 1.0 g/L $(NH_4)_2SO_4$, 0.5 g/L $MgSO_4 \cdot 7H_2O$, 0.1 g/L $FeCl_3 \cdot 6H_2O$, 0.05 g/L $CaCl_2 \cdot 2H_2O$. The strains were cultivated under aerobic conditions at 30℃, 170 rpm in the medium containing 0.15% PHB granules as the sole carbon source. The culture supernatant was applied to a Phenyl-Toyopearl (Tosoh Co., Tokyo, Japan) hydrophobic interaction chromatography column equilibrated with 50 mM Tris-HCl buffer (pH 7.5). Fractions of enzyme with high activity were collected and dialyzed against

distilled water. All the enzyme purification steps were carried out at 4℃.

Enzymatic degradation of PHA granules*:* The activity of PHB depolymerase was assayed spectrophotometrically by measuring the initial decrease in the optical density (O.D.) of the PHA granules at 660 nm. All of the PHA granules were suspended in 50 mM Tris-HCl buffer (pH 8.0) containing 1 mM $MgCl_2$, and their initial absorbance was 3.0 ± 0.03 at 660 nm. The reaction was initiated by the addition of 0.24 μg/mL (final concentration) of purified enzyme suspended in 50 mM Tris-HCl containing 1 mM $MgCl_2$ (pH 8.0). The decrease in turbidity of the PHB polymer was monitored at 660 nm in a shaker bath at 37℃ using a spectrophotometer (Hewlett Packard UV 8452A). As a first order of approximation, the decrease in the turbidity of the PHB granules was analyzed in terms of first-order kinetics, and the degradation rate constant k_1 was calculated [5].

Determination of the enzymatic hydrolysis products. The water-soluble products after complete enzymatic degradation of artificial and native P(HB-co-HV) granules were analyzed using a Shimadzu LC-10A high performance liquid chromatography (HPLC) system equipped with a gradient controller and an SPD-10A UV spectrophotometric detector [6]. The reaction mixtures after enzymatic degradation were harvested by centrifugation (10,000 x g, 10 min) and the clear supernatants were collected and filtered throughout 0.2 μm syringe filter (Minisart, Sartorius Stedim Biotech, Germany) to eliminate residual granule debris. Twenty microliters of the supernatant was injected into the Aminex HPX-87H Ion Exclusion column (300 mm x 7.8 mm, Bio-Rad) [7] and then was eluted with 0.5 mM H2SO4 solution at a flow rate of 0.5 mL/min, 30℃. The water-soluble products were detected at 210 nm. The peaks at 15.94, 18.67, 18.95, 22.06 and 27.40 min were assigned as HB, HB-HB, HV, HB-HV, and HV-HV,

respectively, based on the comparative analysis of the hydrolyzed products of two homopolymers (PHB and PHV) and the four copolymers of known compositions in this study.

Construction and expression of *P. stutzeri* BM190 point mutants. Plasmid isolation, gel electrophoresis, transformation, PCR and cloning for vector construction were performed by standard procedures [8]. An extracellular P(3HB) depolymerase *phaZ* gene was isolated from *P. stutzeri* BM190 and deposited in GenBank under the accession number of EU887946. The *phaZ* gene in P. stutzeri BM190 genomic DNA was PCR-amplified using the primers: PhaZps-BamHⅠ (5'-CATGGATCCATGACCAAGCAATCCTTG-3') which has BamHⅠ site in the translation start codon region and PhaZps-HindⅢ (5'-CCTAAGCTTTCAGTTGCTGCAGCGTCC-3') which has HindⅢ site in the stop codon region. The amplified DNA fragment was cloned into pUC18 vector digested with BamHⅠ and HindⅢ, generating pUC-phaZps. For the construction of point mutants, the desired point mutation primers were designed (5'-CACCGGCCTGTTGTCCGGCGGCG-3' and 5'-CGCCGCCGGACAACAGGCCGGTG-3' for the BM190 phaZ C488T mutation, 5'-GCAGAACAG GAACGCCTGCACCGTGCTGC-3' and 5'-GCAGCACGGTGCAGGCGTTCCTGTTCTGC-3' for the BM190 phaZ A719C mutation) and a PCR was performed using pUC-phaZps as template. The PCR product was digested with DpnI, then transformed into E. coli DH5α. The plasmids (pUC-phaZC488T and pUC-phaZA719C) were purified and the point mutations verified by sequencing. The final rcombinant plasmids were cut with BamHⅠ and HindⅢ, and cloned into pET-28a (+) vector (Novagen, USA). The resulting plasmids pET-PhaZC488T and pET-PhaZA719C expressed His-tagged proteins in E.

coli BL21(DE3) (Novagen, USA). Overnight culture of E. coli BL21(DE3), harboring each recombinant plasmid, was diluted 1:100 in LB medium, After 3 h of cultivation at 37℃, the cultures were induced with 0.1 mM isopropyl-1-thio-β-D-galactopyranoside (IPTG). After 10 h of further cultivation at 25℃, the cells were lysed by sonication (2 kHz, 10 min, 4℃). Whole cells and cell debris were removed by centrifugation for 30 min, 10,000 x g at 4℃. Purification of the His-tagged proteins from the supernatant was performed with NTA chelating agarose CL-6B (Peptron, Korea) according to the manufacturer's instructions.

^{1}H Nuclear Magnetic Resonance Spectroscopy (^{1}H-NMR). The molecular structure of the PHB-1-octadecanol was obtained by ^{1}H NMR with Bruker, DRX-500 MHz spectrometer. The spectra of the samples were recorded at room temperature in $CDCl_3$. The integration of the split spectral signals was performed with standard software.

Differential scanning calorimeter (DSC). Thermal transitions of the end-capped PHB polyesters were measured under nitrogen flow of 50 mL/min by using a differential scanning calorimeter (DSC) (TA Instruments, New Castle, DE), DSC Q200 equipped with a cooling accessory. Samples of 10-15 mg were encapsulated in aluminum pans and heated from -50 to 150℃ at a rate of 10℃/min (first scan). The samples were maintained at 150℃ for 1 min and then cooled to -50℃. They were then reheated from -50 to 150℃ at a rate of 10℃/min (second scan). The melting temperature (T_m) was taken at the peak of the melting endotherm.

Gel permeation chromatography (GPC). GPC measurements were performed with polymer solution in chloroform (1%, w/v), filtered via 0.45 mm Costar Syrfil filters. The molecular weights were determined using an Agilent 1100 series Gel Permeation Chromatography system (Agilent, Santa Clara, CA), consisting of a series of three PLgel columns (105, 103 , and 102), an Agilent G1310A Isocratic pump, an Agilent 1047A RI detector,

an Agilent G1316A column compartment, and an Agilent G1311A vacuum degasser, at a flow rate of 1.0 mL/min. The injection volume of the samples was 60 ml. Chloroform was used as the mobile phase. The run temperature was 30℃. The molecular weight of the polymers were determined relative to polystyrene standards (Polymer Laboratories, Amherst, MA), having a narrow molecular weight distribution.

Determination of particle size. Particle sizes and size distributions were determined by the light scattering method (DLS-8000; Otsuka Electronics Co., Osaka, Japan). The mean particle size of the PHA granules was determined in triplicate and the average values were calculated.

Determination of remaining fructose and NH_4^+. Remaining fructose was determined using 3,5-dinitrosalicylic acid (DNS) method [9]. One milliliter of sample was taken from the supernatant and diluted 50-100 fold. The diluted sample was transferred into a test tube, and was added with 3 ml of DNS reagent. The test tube containing 4 ml mixture was placed in the boiling water bath for 5 min and cooled to room temperature. The absorbance of this reaction mixture was measured at 550 nm (A_{550}) on an X-ma 1000 UV/VIS spectrophotometer (Human Science, Korea). Then, five 1 ml standards containing 0.2, 0.4, 0.6, 0.8 and 1.0 mg of fructose, respectively were prepared according to the same procedure as in the above sample solution. The standard curve obtained showed a linear relation between the absorbance and fructose concentration over the concentration range of 0.1-1.0 mg/mL fructose. As the absorbance of the samples were compared to that of the standard curve, the amount of remaining fructose in the sample was determined.

Remaining NH_4^+ was measured with Nessler's reagent [10]. Nessler reaction was performed by adding 1 mL of Nessler's reagent to 1 mL of the culture supernatant samples. The mixed reaction solution was stirred well, and kept standing for at least 10 min at room temperature. The

absorbance of the reaction mixture was measured at 450 nm (A_{450}). From the standard curve showing a linear relation between the absorbance and NH_4^+ concentration over the concentration range of 0.01-0.03 mg/mL NH_4^+ solution, the absorbance of samples was compared to that of the standard curve and then the amount of remaining NH_4^+ in the sample was determined.

Determination of PHB molecular weight. PHB molecular weight was calculated from the Mark-Howink equation which gives a relation between molecular weight and intrinsic viscosity. The intrinsic viscosities at 30˚C for various PHB samples in chloroform were measured in a capillary viscometer of the Cannon-Fanske type (capillary No. 50) which was immersed in a constant temperature bath. About 0.05 g of sample was dissolved in 10 mL of chloroform and filtered. The efflux time was measured for each concentration (3.0, 2.0, 1.5 and 1.0 mg/mL) of the sample which was prepared by successive dilution. From the calculated specific viscosity, η_{sp} at each concentration, the zero concentration-extrapolated intrinsic viscosity [η] was obtained to calculate the PHB molecular weight from the following Mark-Howink equation: $[\eta] = kM^a$ where, k, 7.7 x 10^{-5} (cm^3 g^{-1}) and a, 0.82 [11].

[1] Aldor I, Keasling JD. 2001. Metabolic engineering of poly(3-hydroxybutyrate-*co*-3-hydroxyvalerate) in recombinant *Salmonella enterica* serovar Typhimurium. Biotechnol Bioeng 76:108-114.

[2] Aldor IS, Kim SW, Prather KLJ, Keasling JD. 2002. Metabolic engineering of a novel propionate-independent pathway for the production of poly(3-hydroxybutyrate-*co*-3-hydroxyvalerate) in recombinant *Salmonella enterica* serovar Typhimurium. Appl Environ Microbiol 68:3848-3854.

[3] Choi MH, Song JJ, Yoon SC. 1995. Biosynthesis of copolyesters by *Hydrogenophaga pseudoflava* from various lactones. Can J Microbiol 41(Suppl. 1):60-67.

[4] Choi MH, Yoon SC. 1994. Polyester biosynthesis characteristics of *Pseudomonas citronellolis* grown on various carbon sources, including 3-methyl-branched substrates. Appl Environ Microbiol 60:3245-3254.

[5] S.C. Yoon, M.H. Choi, Local sequence dependence of polyhydroxyalknoic acid degradation in *Hydrogenophaga pseudoflava*, J. Biol. Chem. 274 (1999) 37800-37808.

[6] Macedo AC, Tavares TG, Malcata FX. 2003. Esterase activities of intracellular extracts of wild strains of lactic acid bacteria isolated from Serra da Estrela cheese. Food Chemistry 81:379-381.

[7] Karr DB, Waters JK, Emerich DW. 1983. Analysis of poly-β-hydroxybutyrate in *Rhizobium japonicum* bacteroids by ion-exchange high-pressure liquid chromatography and UV detection. Appl Environ Microbiol 46:1339-1344.

[8] Sambrook J, Russel DW. 2001.Molecular cloning: A laboratory manual. 3rd Ed. New York: Cold Spring Harbor Laboratory Press.

[9] Miller GL. 1959. Use of dinitrosalicylic acid reagent for determination of reducing sugars. Anal Chem 31:426-428.

[10] Fischer RB, Peters DG. 1968. Quantitative chemical analysis (Philadelphia: W.B. Saunders Co.), pp. 677-679.

[11] Marchessault RH, Okamura K, Su CJ. 1970. Physical properties of poly(β-hydroxybutyrate). Ⅱ. Conformational aspects in solution. Macromolecules 3:735-740.

Supplementary Figure S1. Characterization of synthesized PHB-1-octadecanol

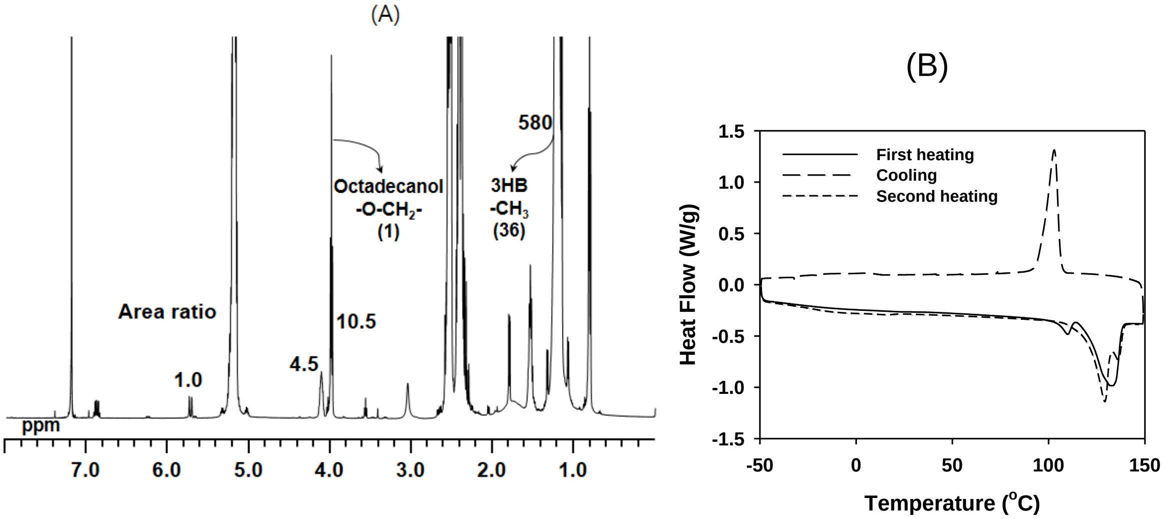

Supplementary Figure S1. Characterization of synthesized PHB-1-octadecanol: (A) ^{1}H NMR analysis for the calculation of the degree of esterification and the number average molecular weight (Mn). The absorption peak at 3.99 ppm (area ratio = 10.5) is associated with the triplet methylene proton in the terminal alcohol group forming an ester linkage in the connecting region between PHB and 1-octadecanol. The strong absorption peak at 1.21 ppm (area ratio = 580) is for the protons in the methyl group in 3HB-units. Two minor absorption peaks at 6.88 and 5.73 ppm (area ratio = 1.0 equally) are associated with the olefinic end group resulting from thermal dehydration of the hydroxyl terminal group. The minor absorption at 4.11 ppm (area ratio = 4.5) is related to the methine proton (CH_3-C*H*(OH)-) in the terminal hydroxyl containing group. NMR peak ratio analysis showed that in the purified PHB-1-octadecanol sample, ~20% of the hydroxyl groups was replaced by alkenic proton and ~80% of free hydroxyl group was retained. The fact that the sum of the peak areas for the olefinic proton and methine proton in the opposite end of PHB are almost equal to the half of the peak area at 3.99 ppm peak ascribing to the hydroxyl adjoining methylene in 1-octadecanol revealed that more than 98% of the terminal carboxyl groups were esterified with 1-octadecanol. Mn was calculated to be ~3000 from ^{1}H-NMR data

(the mole ratio of 1-octadecanol and HB-unit was 1:36). (B) Thermal transition analysis. The T_m value of PHB-1-octadecanol was observed at ~130℃. Dried PHB-1-octadecanol powder exhibited the same crystalline peak pattern as high PHB homopolymer[1]. Thus, the end-group capping did not induce any significant kink or deformation to perturb the folding of PHB for crystallization.

Supplementary Figure S2. HPLC analysis of the enzymatic degradation of PHB granules to find any small calcium chelating ligand molecule

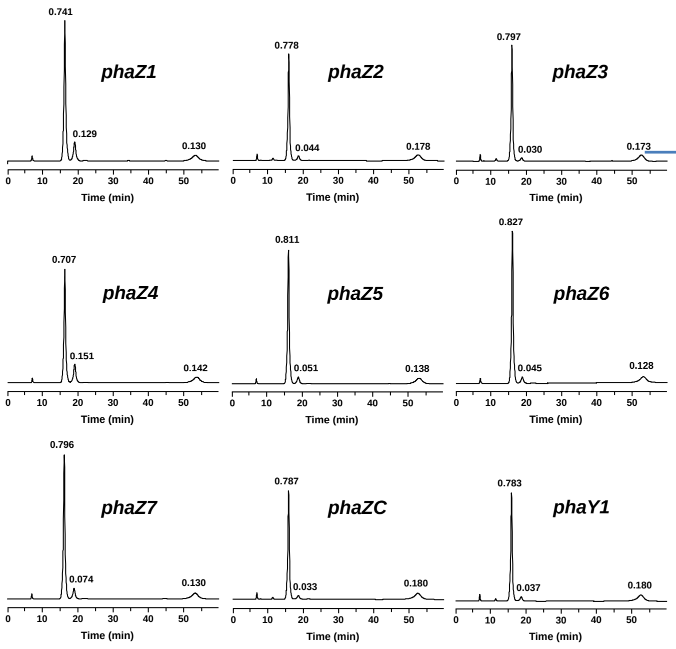

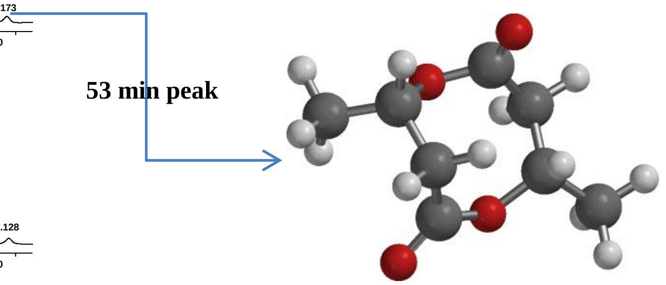

HPLC analysis of the enzymatic degradation products of native PHB nanogranules accumulated in various *phaZ* mutants

Name	Function	PHA Depolymerase Superfamily designation	Protein ID	Locus tag	Degradation Product
**PhaZ1**	Intracellular PHA depolymerase	i-nPHAscl (no lipase box)	CAJ92291.1	H16_A1150	Oligomer and monomer
**PhaZ2**	Intracellular PHA depolymerase	i-nPHAscl (no lipase box)	CAJ95139.1	H16_B0339	Unknown
**PhaZ3**	Putative intracellular PHA depolymerase	i-nPHAscl (no lipase box)	CAJ93939.1	H16_A2862	Monomer
**PhaZ4**	Putative PHA depolymerase	i-nPHAscl (no lipase box)	AAP85930.1	PHG178	Unknown
**PhaZ5**	Intracellular PHA depolymerase	i-nPHAscl (no lipase box)	CAJ95805.1	H16_B1041	Unknown
**PhaZ6**	PHA depolymerase	e-dPHAscl (catalytic domain type 1)	CAJ96855.1	H16_B2073	Dimer and trimer
**PhaZ7**	PHA depolymerase	e-dPHAscl (catalytic domain type 1)	CAJ97183.1	H16_B2401	Unknown
**PhaY1**	D-(-)-3-hydroxylbutyrare oligomer hydrolase	Unknown	CAJ93348.1	H16_A2251	Monomer
**PhaZC**	D-(-)-3-hydroxylbutyrare oligomer hydrolase	Unknown	CAJ92475.1	H16_A1335	Monomer

The data in the table was cited from Brigham et al. AMB Express 2012, 2:26.and modified .

HPLC analysis of the enzymatic degradation products of artificial PHB and PHV nanogranules

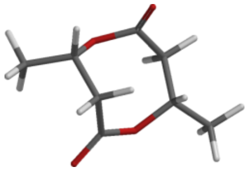

Cyclic 3HB dimer

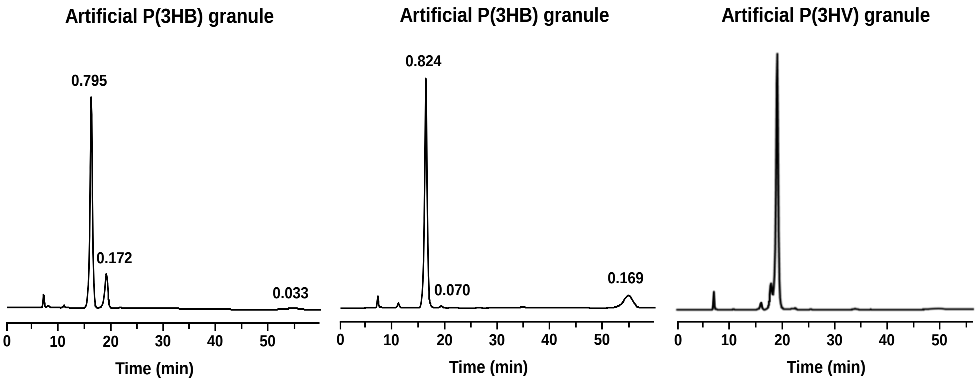

The absence of the 53 min-peak for P(3HV) granules from *Paracoccus denitrificans* (Ca^{2+} content: 3.13 mg/g PHV) →→ produced as a side product in the enzymatic degradation.

Supplementary Figure S2. HPLC analysis of the enzymatic degradation products displayed only the PHB hydrolysis products (monomer, dimer and cyclized dimer against P(3HB) native granules isolated from 7 *phaZ* mutants and the other two mutants of oligomer hydrolases, *phaY1* and *phaZC*). The cyclized 3HB dimer was initially assumed as a Ca^{2+} chelating ligand. But only 3HV monomer and dimer was detected and the peak at 53 min was not observed in the degradation of P(3HV) artificial granules with BM190 WT enzyme even though native P(3HV) granules isolated from *Paracoccus denitrificans* contained 3.138 mg Ca^{2+} /g PHA. Therefore, we concluded that the tiny amount of cyclic 3HB dimer appeared at 53 min was ascribable to a byproduct of the enzymatic degradation reaction of PHB and it was excluded from a candidate molecule for Ca^{2+} chelating ligand in our assumption.

Supplementary Figure S3. Comparative analysis of the five selected divalent cations sorption data

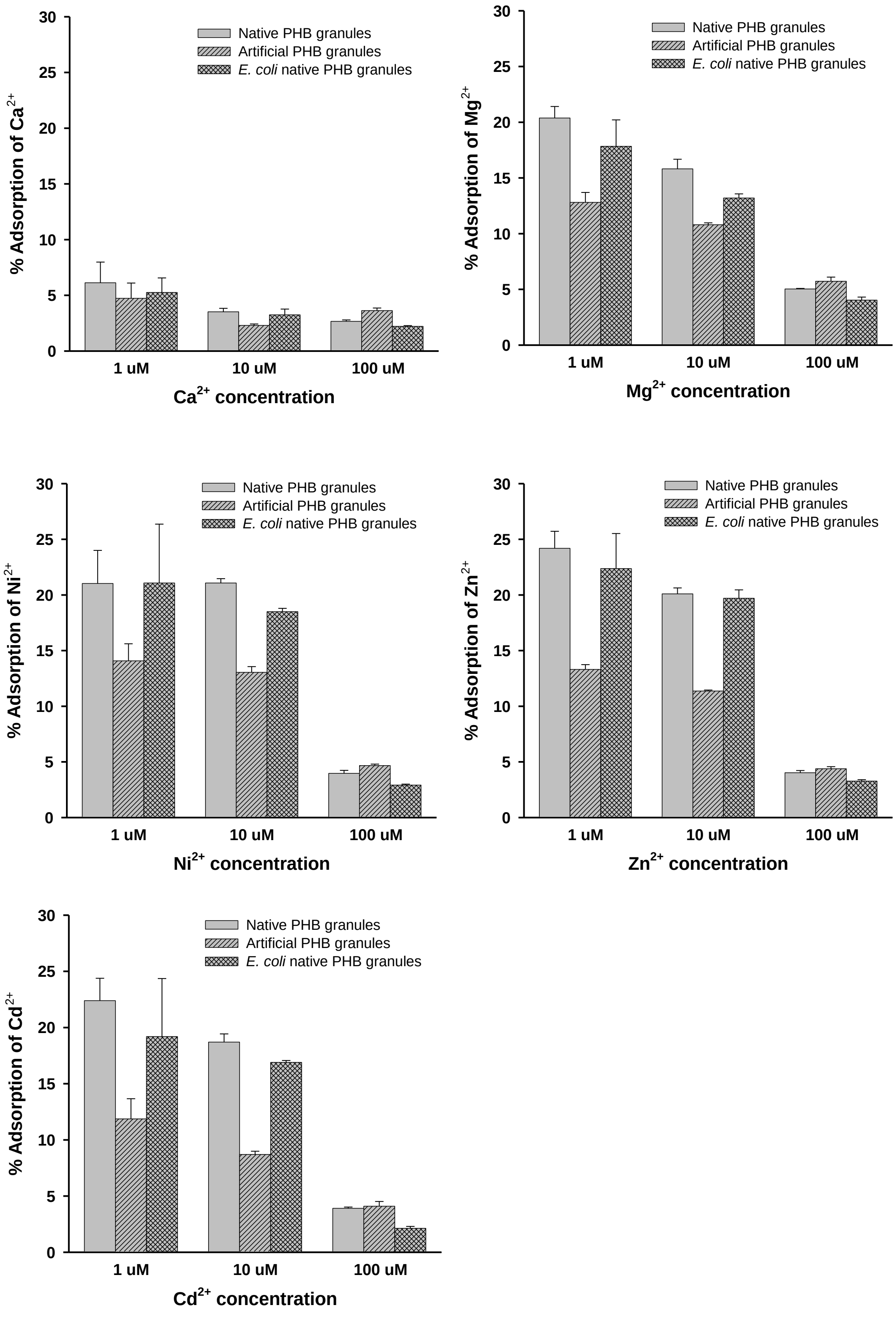

Supplementary Figure S3. The percentage adsorption of five divalent metal ions including Ca^{2+} , Mg^{2+}, Ni^{2+}, Cd^{2+} and Zn^{2+}, against the nanogranules, native, artificial and *E. coli* native granules depending on their concentrations.

Supplementary Figure S4A. X-ray diffraction data for freeze-dried PHB granules.

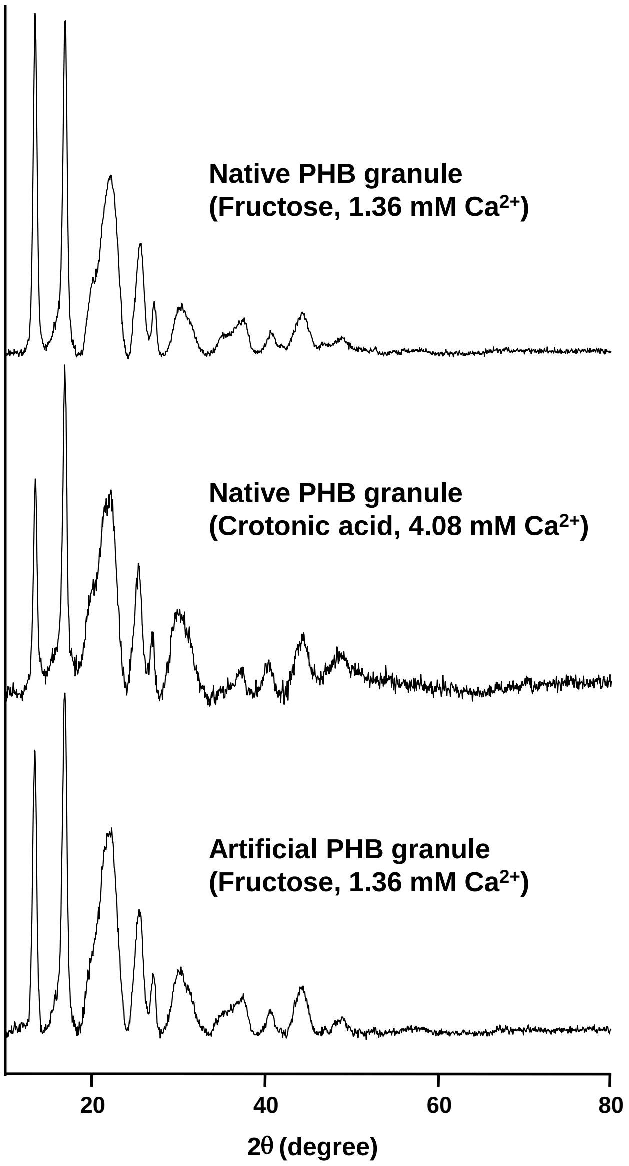

Supplementary Figure S4A. X-ray diffraction profiles of freeze-dried PHB granules. The native granules from crotonic acid ($[Ca^{2+}]_{ex}$ = 4.08 mM) grown H16 cells had 15 times more amount of calcium ion than that from fructose grown cells but the profile patterns are not significantly different among all PHB granules independent of Ca^{2+} level in the samples. This may indicate that Ca^{2+} ions are localized in the core storage site so that the core Ca^{2+} ions do not disturb the overall dimension of the polymer chain folding.

Supplementary Figure S4B. Powder FTIR analysis for freeze-dried PHB granules.

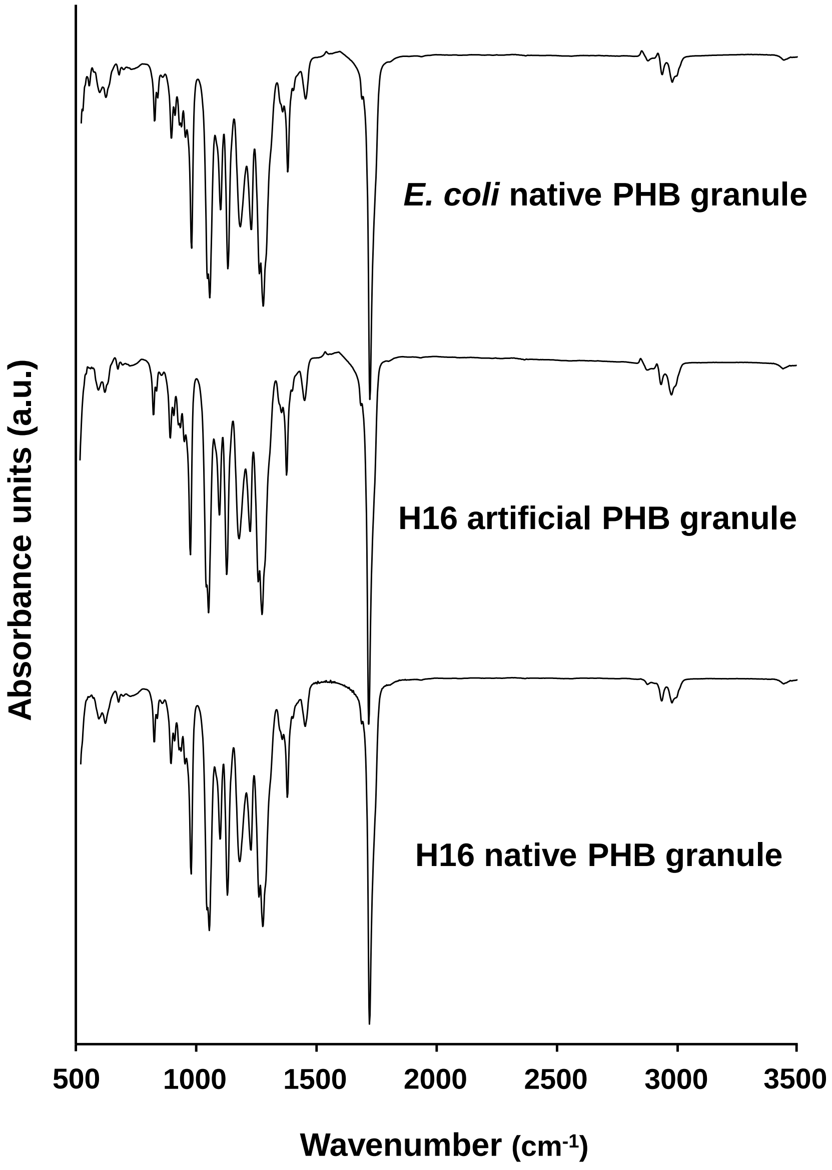

Supplementary Figure S4B. FTIR spectra of freeze-dried H16 and *E. coli* native PHB granules and artificial PHB granules. The absorption spectra were obtained by irradiating the powder sample without KBr pelleting. The spectra of them showed almost the same absorption intensity at every absorption peak. Thus, any Ca^{2+} effect on the spectrum was not observed. Similar to X-ray diffraction data, the similar IR absorptivity of the PHB granules may indicate that Ca^{2+} ions are localized in a storage site.

Supplementary Table S1. Primers of PHA synthase and depolymerases related genes for insertional mutants

Name	sequence	Restriction site	Length (bp)
phaC	F: TCCCCCGGGTACGGTGTTTCTGGTGTCGT	SmaI	435
	R: CTAGTCTAGATTGCCCTTCAGGTAGTTGTC	XbaI	
phaZ1	F: TCCCCCGGGTCGTGGCGATGAACCCG	SmaI	391
	R: GCTCTAGACCCGAGAAGATGCCGTAGTG	XbaI	
***phaZ*2**	F: CGGAATTC CACGCATCTGGTGGCGATTT	EcoR I	430
	R: GCTCTAGA CAGGCTGACGGTTTCCAGGTAG	XbaI	
***phaZ*3**	F: CGGAATTCCTGCGGTTTCCCTGTTGGC	EcoRI	465
	R: GCTCTAGACGGTGGTACGGATGTCCTGTG	XbaI	
***PhaZ*4**	F: CGGAATTCAGTGGTTCCGACGCAATCTG	EcoRI	461
	R: GCTCTAGA TACAACCGGCAATCGTGACC	XbaI	
***phaZ*5**	F: CGGAATTC AGATGGCCGAAGACAACGAG	EcoRI	395
	R: GCTCTAGA CAGGTGATGCTCCAGGAAAACC	XbaI	
***phaZ*6**	F: CGGAATTCCGGAAGCCCAGGTGAAGTCC	EcoRI	346
	R: GCTCTAGATGCGGGTAGGCCACGATAAA	XbaI	
***phaZ*7**	F: CGGAATTCCGCACTGTCGGGCGTGGTAT	EcoRI	568
	R: GCTCTAGACTGCATGGCCTTGGCATCG	XbaI	
***phaZ*c**	F: CGGAATTCCGTGGAGAAGGTGGACTGGTTC	EcoRI	354
	R: GCTCTAGAGGCGTGGATTTGCGGAAC	XbaI	
PhaY1	F: CGGAATTCGTCGTTCAACACCTCGAAGCC	EcoRI	377
	R: GCTCTAGATGATCGCCCAGATCGCAAAT	XbaI	

Supplementary Table S2. Particle size of artificial (non-end capped) PHA granules (reassembled using the PHA chloroform-extracted from cells) and PHB-1-octadecanol which were prepared by chloroform/water emulsion technique) and number average molecular weight of the polymers

Type of polymer (Bacterium used for synthesis)	Number-average molecular weight of polymer [a]	Particle size of artificial granules [c] (dia. (nm))
**PHB (*C. necator* H16)**	369,700	230±20
**PHV (*P. denitrificans*)**	Not determined	238±4
**P(75 mol% HB-co-25 mol% HV) (*H. pseudoflava*)**	16,600	249±9
**P(65 mol% HB-co-35 mol% HV) (*H. pseudoflava*)**	73,200	224±7
**P(42 mol% HB-co-58 mol% HV) (*H. pseudoflava*)**	68,300	223±3
**PHB-1-octadecanol**	3,000[b]	158±33

[a], Determined by gel permeation chromatography.

[b], Calculated from H-NMR data.

[c], Determined by light scattering method.

Supplementary Table S3. $[Ca^{2+}]_{ex}$ dependence of PHA accumulation in other soil bacteria.

Strains	Carbon source (mM)	Type of PHA	PHA content (wt%)		Amount of Ca^{2+} (mg/g PHA)[a]
			0 mM Ca^{2+}	1.36 mM Ca^{2+}	
Hydrogenophaga pseudoflava	Glucose (70)	P(3HB)	7.5±2.1	63.7	3.54
Paracoccus denitrificans	Valeric acid (92)	P(3HV)	5.6	31.0	3.14
Pseudomonas putida	Octanoic acid (40)	P(3HAs)[b]	3.8	27.0	2.85
Pseudomonas fluorescens	Fructose (70)	P(3HAs)[c]	2.3	25.2	2.79
	Octanoic acid	P(3HAs)[d]	0.8	23.3	2.95
Pseudomonas aeruginosa	Fructose (70)	P(3HAs)[e]	0.4	12.6	2.33
	Octanoic acid	P(3HAs)[f]	1.9	28.5	3.10
Pseudomonas aeruginosa	Fructose (70)	P(3HAs)[g]	0.7	14.8	2.46
	Octanoic acid	P(3HAs)[h]	2.7	25.8	2.76

[a] data for native PHA granules isolated from bacteria grown on 1.36 mM Ca^{2+}.

[b] P(3-hydroxyhexanoate (3HH)-co-3-hydroxyoctanoate (3HO)-co-3-hydroxydecanoate (3HD)) copolymer composed of 3HO as major monomer.

[c] P(3HO-co-3HD-co-3-hydroxydodecanoate (3HDD)-co-3-hydroxydodecenoate ($C_{12:1}$)-co-3-hydroxytetradecanoate-co-3-hydroxytetradecenoate) copolymer composed of 3HO, 3HDD, and $C_{12:1}$ as major comonomers.

[d] P(3HH-co-3HO-co-3HD) copolymer composed of 3HO as major monomer.

[e] P(3HH-co-3HO-co-3HD) copolymer composed of 3HO and 3HD as major comonomers.

[f] P(3HH-co-3HO-co-3HD) copolymer composed of 3HO as major monomer.

[g] P(3HH-co-3HO-co-3HD) copolymer composed of 3HO and 3HD as major comonomers.

[h] P(3HH-co-3HO-co-3HD) copolymer composed of 3HO as major monomer.

Supplementary Table S4. Determination of the amounts of Ca^{2+} (mg/g PHB) in various *Cupriavidus necator* H16 *phaZ* gene mutants which were grown on 20 g/L fructose in M1 medium in the presence of 1.36 mM Ca^{2+}.

**Strains**[1]	**Function**	**Dry cell weight (g/L)**	**PHB weight (g/L)**	**Amount of Ca^{2+} (mg/g PHB)**
**PhaZ1**	Intracellular PHA depolymerase	7.09	5.09	2.98
**PhaZ2**	Intracellular PHA depolymerase	7.05	5.05	3.21
**PhaZ3**	Putative intracellular PHA depolymerase	6.98	4.98	3.07
**PhaZ4**	Putative PHA depolymerase	7.12	5.04	2.57
**PhaZ5**	Intracellular PHA depolymerase	6.90	5.01	3.11
**PhaZ6**	PHA depolymerase	6.86	4.89	3.15
**PhaZ7**	PHA depolymerase	7.14	4.94	3.04
**PhaZC**	D-(-)-3-hydroxybutyrate oligomer hydrolase	7.13	5.13	3.13
**PhaY1**	D-(-)-3-hydroxybutyrate oligomer hydrolase	7.20	5.15	3.27

[1], Brigham *et al*, Examination of PHB depolymerases in *Ralstonia eutropha*: Further elucidation of the roles of enzymes in PHB homeostasis. *AMB Express* 2012, **2**:26. (doi:10.1186/2191-0855-2-26).

The above data showed that the genes related with intracellular PHB degradation have no effect on Ca^{2+} controlled PHB accumulation.

Supplementary Table S5. Elemental analysis of hypochlorite-treated and recovered P(3HB) granules

	C<br>wt%[a)]	H<br>wt%	O<br>wt%	N<br>wt%	S<br>wt%	P[c)]<br>μg/gPHB
Sample	54.46	6.49	38.26[b)]	0.762	0.031	6
Theoretical	55.81	6.98	37.21	-	-	

[a)] The values are averages of four measurements (Elemental Analyzer).

[b)] The undetermined deficit in the elemental analysis was assumed to be due to oxygen atom.

c) ICP-AES analysis method was used.
